# Oxetanes: formation, reactivity and total syntheses of natural products

**DOI:** 10.3762/bjoc.21.101

**Published:** 2025-06-27

**Authors:** Peter Gabko, Martin Kalník, Maroš Bella

**Affiliations:** 1 Institute of Chemistry, Slovak Academy of Sciences, Dúbravská cesta 9, 845 38 Bratislava, Slovakia

**Keywords:** medicinal chemistry, natural products, oxetane, reactivity, synthesis

## Abstract

Oxetanes are 4-membered cyclic monoethers which have found important applications in medicinal chemistry as polar and metabolically stable isosteric replacements for *gem*-dimethyl and carbonyl groups. This work reviews possible synthetic strategies towards these strained heterocycles, covering both de novo constructions of the 4-membered ring as well as derivatisations of oxetane building blocks, then reactivity of oxetanes in terms of ring-opening and ring-expansion reactions, and finally total syntheses of selected oxetane-containing natural products. The literature review primarily covers reports made after the year 2015, but a few older contributions that were considered relevant are also discussed.

## Introduction

**Figure 1:**
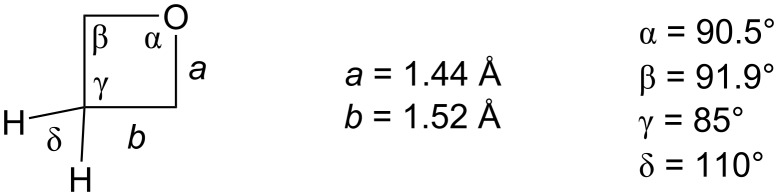
Bond lengths and bond angles in oxetane at 140 K [2].

Oxetanes are 4-membered heterocyclic compounds containing one oxygen atom whose discovery dates back to the 1870s when the first synthesis of the parent, unsubstituted oxetane was reported by Reboul [1]. Over the next 100 years, it was generally accepted that this cyclic ether is planar but this myth was eventually debunked by Luger and Buschmann in 1984 who carried out the first X-ray analysis of oxetane [2] and calculated a small puckering angle of 8.7° at 140 K, which is much smaller than the approximate 30° puckering angle in cyclobutane [3] – this difference is most likely caused by fewer gauge interactions in oxetane where one of the methylene units is replaced by oxygen. The authors also reported bond angles and lengths which are shown in Figure 1.

As expected, the endocyclic angles are far from the ideal tetrahedral value which results in a large ring strain of 25.5 kcal/mol, comparable to oxirane (27.3 kcal/mol) and much greater than tetrahydrofuran (5.6 kcal/mol) [4]. Moreover, the strained C–O–C bond angle effectively exposes the oxygen lone pairs, making oxetane a strong hydrogen-bond acceptor and Lewis base [5]. In fact, its hydrogen-bond-accepting ability is even stronger than that of the other 3-, 5- and 6-membered cyclic ethers, as well as the carbonyls of aldehydes, ketones, esters and carbonates [6–8]. The only better carbonyl-based hydrogen-bond acceptors are amides, carbamates and ureas [9].

**Figure 2:**
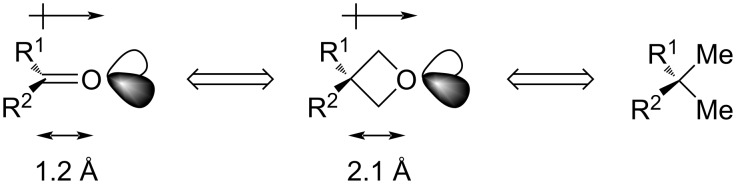
Analogy of 3-substituted oxetanes to carbonyl and *gem*-dimethyl groups [12].

The first to recognise that these unique physicochemical properties may have valuable applications in medicinal chemistry were Carreira, Rogers-Evans, Müller and colleagues, who together reported that 3-substituted oxetanes can serve as isosteric replacements for carbonyl and *gem*-dimethyl groups (Figure 2) [10–12].

As for the analogy to carbonyls, oxetanes display comparable polarity, spatial arrangement of the oxygen lone pairs and hydrogen-bond-accepting ability. In addition, they possess one major advantage and that is greater metabolic stability: while carbonyl groups are susceptible to additions and hydrolyses (in case of esters and amides), as well as epimerisations at the α-carbons, oxetanes are stable to such chemical transformations. In case of the *gem*-dimethyl group, which is commonly used to block metabolically labile sites at the expense of raising lipophilicity, bridging the two methyls with an oxygen atom effectively eliminates its lipophilic character and reduces susceptibility to metabolic attack, while practically maintaining the molar volume.

**Figure 3:**
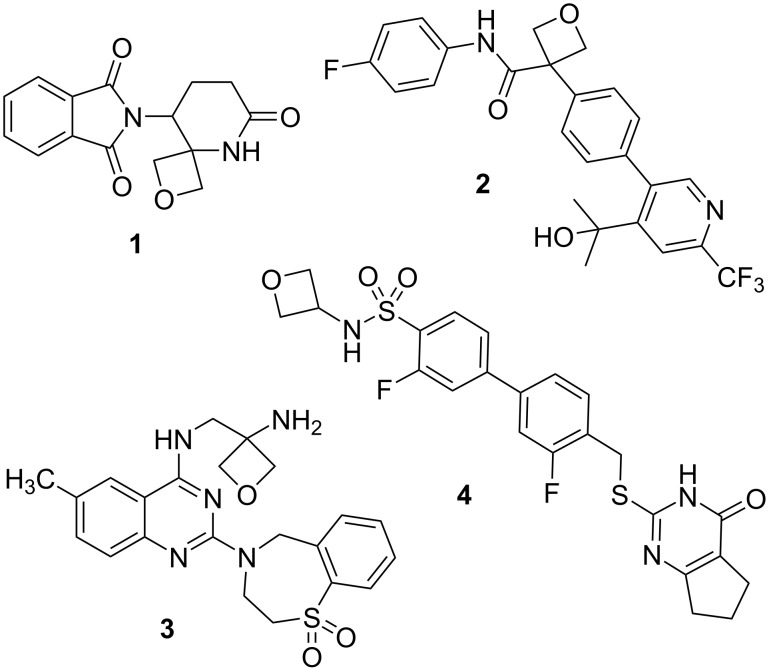
Use of oxetanes in drug design – selected examples.

Because controlling polarity and metabolic stability is a major issue in medicinal chemistry, 3-substituted oxetanes have eventually become quite attractive structural motifs in drug design [13–14]. A few examples are shown in Figure 3: oxetano-thalidomide **1** was designed as an analogue of the infamous thalidomide to block racemisation and hence prevent the severe side-effects caused by the opposite enantiomer [15]. Compound **2** is a highly cytotoxic agent inhibiting IDO1 (indole-amine 2,3-dioxygenase) which possesses an excellent pharmacokinetic profile and is suitable for both oral and parenteral dosing [16]. Compound **3**, called ziresovir, is a promising candidate for treatment of the respiratory syncytial virus (RSV) infection in infants and it has successfully completed a phase III clinical trial [17–18]. Finally, sulphonamide **4** is a lead compound for the treatment of osteoarthritis via MMP-13 (matrix metalloproteinase 13) inhibition which exhibited an excellent selectivity profile and complete inhibition of collagenolysis in vitro [19].

**Figure 4:**
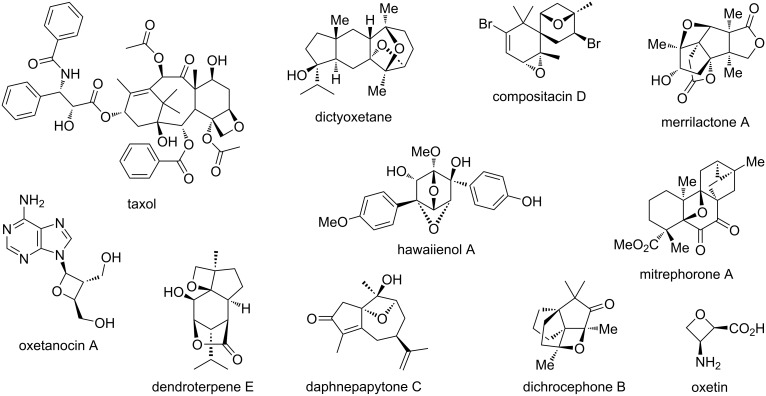
Examples of oxetane-containing natural products.

As for natural occurrence of oxetanes, they are relatively uncommon, and an extensive review has recently been published by Dembitsky which explored the structural features and biological activities of oxetane-containing natural products [20]. Some of the most famous examples are taxol [21], oxetanocin A [22], oxetin [23], merrilactone A [24], dictyoxetane [25] and mitrephorone A [26], which were isolated by 2005, but the collection is still being expanded and more recent additions include dichrocephone B from 2013 [27], compositacin D from 2017 [28], hawaiienol A from 2018 [29] or dendroterpene E [30] and daphnepapytone C [31] from the 2020s (Figure 4). Most of these compounds possess intriguing biological activities and selected examples from this list are discussed in more detail in chapter 4 with regards to their isolation, bioactivity and a recent total synthesis.

The aim of this work is to provide a comprehensive review of oxetanes in terms of their preparation and synthetic utility. Because multiple reviews of similar formats covering literature up to late 2015 have already been published [12,32–34], we will discuss primarily the most recent advancements since the year 2016 complemented by a few older works that were deemed powerful and relevant for illustrating a given reaction mode. Chapter 1 covers strategies for the oxetane ring formation including substitutions, cycloadditions, ring-size manipulations and carbene insertions. Chapter 2 details chemical transformations of 3-oxetanone leading to advanced oxetane building blocks. In chapter 3, we review the reactivity of oxetanes with regards to ring openings and ring expansions including both symmetric and enantioselective variants. Finally, chapter 4 covers isolations, biological activities and total syntheses of selected oxetane-containing natural products with a focus on the most critical and strategic bond formations.

## Review

### 1 Construction of the 4-membered ring

**Scheme 1:**
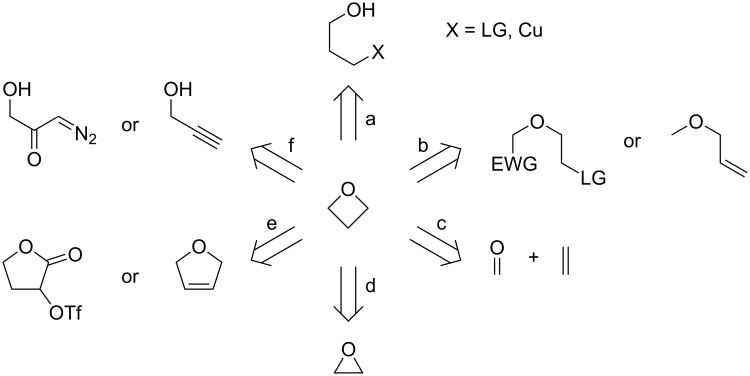
Synthetic strategies towards construction of the oxetane ring.

One of the two principal approaches towards the synthesis of oxetanes is constructing the 4-membered ring de novo. This can be further categorised into 6 synthetic strategies as depicted in Scheme 1: a) C–O bond-forming cyclisations, b) C–C bond-forming cyclisations, c) [2 + 2] cycloadditions between carbonyls and alkenes, d) ring expansions, e) ring contractions and f) O–H insertions. In the following subchapters, these strategies will be discussed in more detail and illustrated by specific examples.

#### 1.1 C–O bond-forming cyclisations

**Scheme 2:**
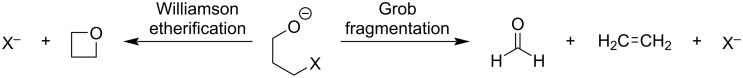
Overview of intramolecular Williamson etherification and competing Grob fragmentation.

**1.1.1 Intramolecular Williamson etherifications:** Discovered by Alexander Williamson in 1850, this reaction is an S_N_2 substitution in which a leaving group, typically a halide or sulphonate ester, is displaced by an alkoxide anion producing an ether (Scheme 2) [35]. Although this seems to be a relatively simple, straightforward reaction, the yields are often low due to the competing Grob fragmentation (Scheme 2) which, besides being entropically favoured, might also be favoured by the thermodynamic stability of the resulting alkene [36]. In addition, this intramolecular etherification corresponds to the 4-*exo*-*tet* cyclisation which, in terms of kinetics, is the least favoured n-*exo*-*tet* cyclisation mode where *n* ≤ 7 [37]. Nevertheless, the Williamson etherification still remains one of the most common methods for oxetane synthesis, mainly due to its practicality and versatility.

**Scheme 3:**
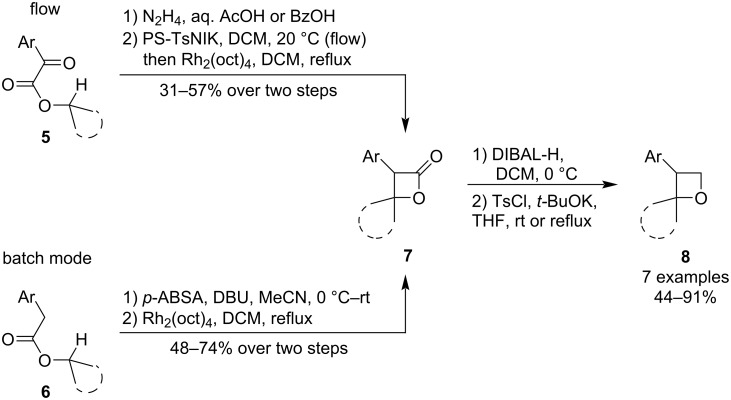
Synthesis of spiro-oxetanes via 1,4-C–H insertion and Williamson etherification.

*1.1.1.1 Substitution of a leaving group:* In 2017, Moody et al. developed a new route towards spiro-oxetanes **8** utilising a combination of 1,4-C–H insertion and Williamson etherification (Scheme 3) [38]. The methodology commences from esters of functionalised arylacetic acid **5** or **6** and involves two separate protocols: first, a metallacarbene, which undergoes the insertion, is generated from the corresponding diazo precursor formed either in flow via hydrazone oxidation (PS-TsNIK packed column), or in batch mode via diazo transfer. In the second protocol, the β-lactone intermediates **7** are reduced to a diol and a subsequent Williamson etherification affords the oxetanes. Since both protocols consist of two steps and give only moderate yields, the overall oxetane yield and synthetic efficiency are rather low.

**Scheme 4:**
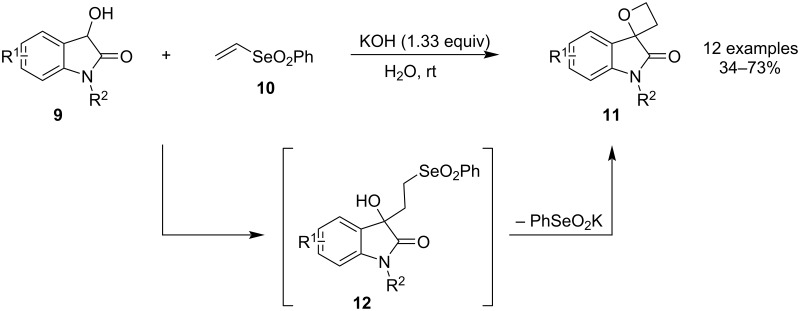
Use of phenyl vinyl selenone in the synthesis of spirooxindole oxetanes.

In 2019, Marini and colleagues published a one-step synthesis of spirooxindole 2,2-disubstituted oxetanes **11** via an unprecedented addition/substitution cascade (Scheme 4) [39]. The protocol reacts readily available 3-hydroxyindolinones **9** with phenyl vinyl selenone (**10**) in aqueous KOH at room temperature and gives moderate to good yields of the spirocycle. The reaction is assumed to proceed through a Michael addition followed by Williamson etherification of the resulting γ-hydroxyselenone **12**.

**Scheme 5:**
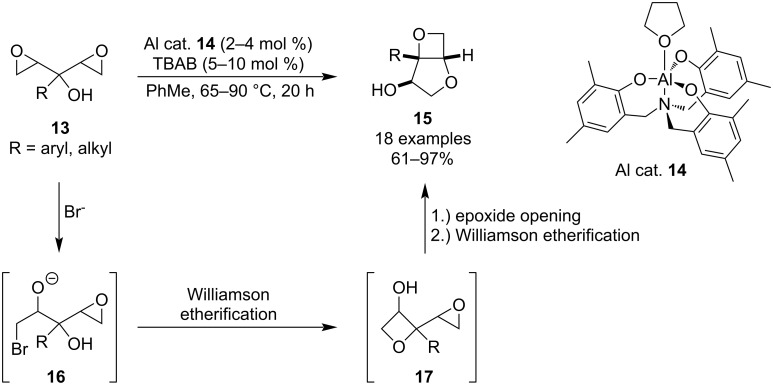
Synthesis of bicyclic 3,5-anhydrofuranoses via double epoxide opening/etherification.

In 2022, Kleij et al. reported a domino synthesis of bicyclic 3,5-anhydrofuranoses **15** using easily accessible bis-epoxy alcohols **13** and a binary Al/TBAB catalyst (Scheme 5) [40]. The reaction is carried out in toluene upon mild heating, providing the bicyclic products in high to excellent yields. Both electron-rich and electron-poor phenyls as well as aliphatic chains worked well, however, increased temperature and catalyst loadings were necessary for *ortho*-substituted phenyls. Control experiments and DFT calculations revealed that the oxetane ring is formed before the tetrahydrofuran in the domino process.

**Scheme 6:**
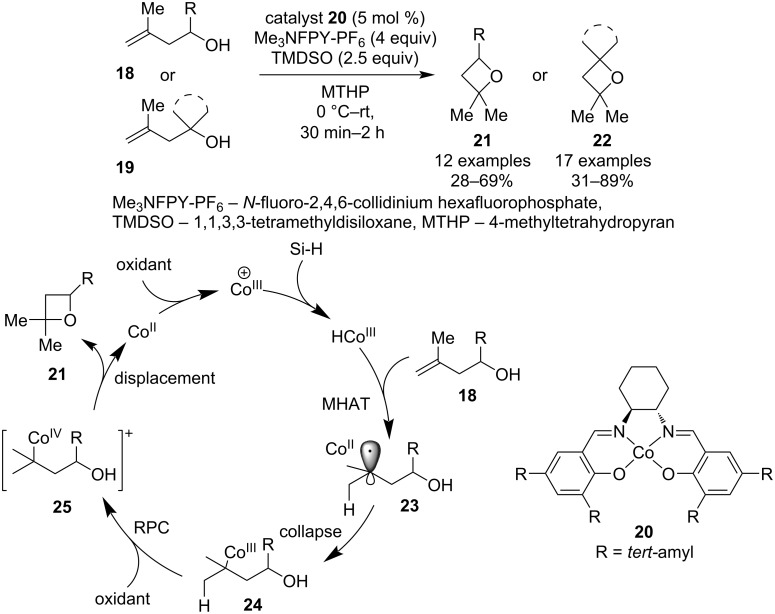
Preparation of spirooxetanes by cycloisomerisation via MHAT/RPC.

In 2023, Shigehisa and co-workers published a new cycloisomerisation strategy for the construction of oxetane rings from homoallylic alcohols **18**/**19** via metal hydride atom transfer/radical polar crossover (MHAT/RPC) method (Scheme 6) [41]. This mild and high-yielding protocol displays good functional group tolerance and has a broad substrate scope, even providing access to medicinally relevant spirooxetanes. The proposed MHAT/RPC mechanism starts with a single-electron oxidation of the cobalt catalyst followed by a reaction with the siloxane to generate a cobalt–hydride complex. Subsequent hydride transfer to the alkene produces radical pair **23** which collapses to alkylcobalt intermediate **24**. Another single-electron oxidation of the metal centre turns the cobalt into an excellent leaving group, allowing for an intramolecular displacement reaction that affords the oxetane ring and regenerates the Co(II) catalyst.

**Scheme 7:**
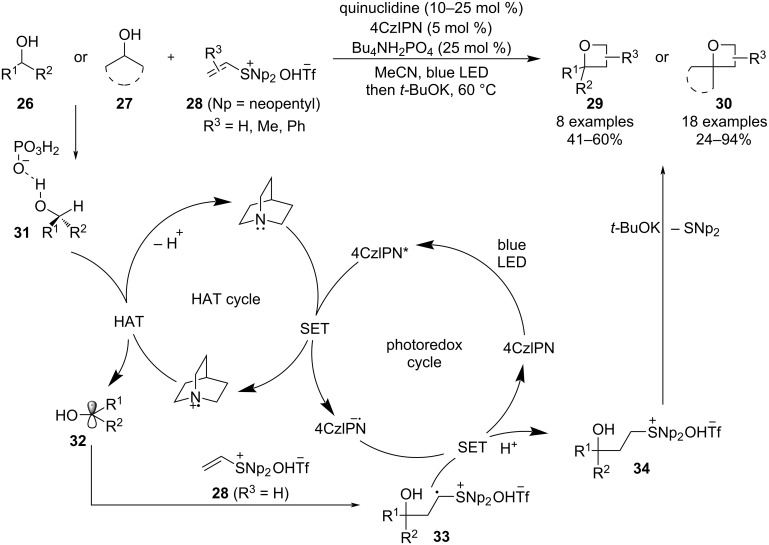
Oxetane synthesis via alcohol C–H functionalisation.

In 2023, Silvi et al. described a versatile and practical methodology that couples Williamson etherification with alcohol C–H functionalisation, thus creating a unique synthetic strategy towards oxetane formation that avoids tedious multistep substrate preparations (Scheme 7) [42]. It can be initiated from simple, unactivated primary or secondary alcohols, tolerates various functional groups such as acetals, amides or esters and occurs under very mild conditions that are also suitable for a late-stage functionalisation of complex molecules. The transformation is based on an H-atom transfer to photochemically oxidised quinuclidine followed by an annulation of the resulting ketyl radical **32** with vinylsulphonium triflates **28** which combine the features of a radical acceptor (in a Giese-type addition) and a leaving group.

**Scheme 8:**
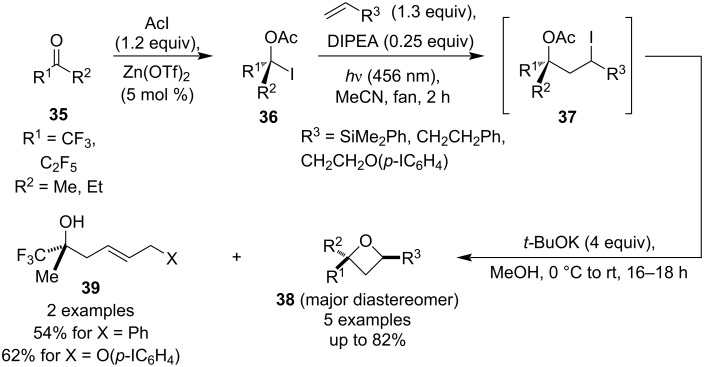
Access to oxetanes **38** from α-acetyloxy iodides.

The use of ketyl radicals for oxetane synthesis was also investigated by Schindler and co-workers and a year later the group published a methodology that utilises a similar Giese addition/Williamson etherification sequence (Scheme 8) [43]. In this case, however, the radicals were generated by irradiation of α-acetyloxy iodides **36**, formed by treating the corresponding ketone precursors with acetyl iodide in the presence of catalytic Zn(OTf)_2_. The radical addition employed electron-rich alkenes and the resulting 1,3-acetyloxyiodides **37** were treated in situ with methanolic *t*-BuOK to promote the cyclisation. Best results for the oxetane formation were obtained for dimethylphenylvinylsilane, while alkenes without the silyl group afforded mainly homoallylic alcohols **39**, presumably through an intramolecular E2 elimination.

**Scheme 9:**
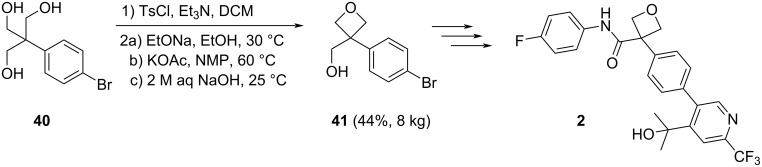
The kilogram-scale synthesis of oxetane intermediate **41**.

Finally, the importance and power of the intramolecular Williamson etherification has also been demonstrated by the kilogram-scale synthesis of oxetane intermediate **41**, which is a key intermediate in the preparation of the previously mentioned IDO1 inhibitor **2** (Scheme 9) [16].

**Scheme 10:**
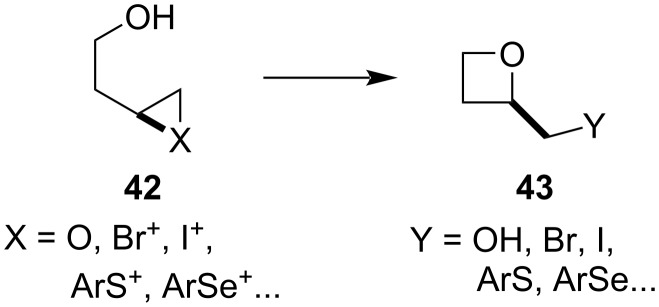
Overview of the intramolecular opening of 3-membered rings.

*1.1.1.2 Opening of a 3-membered ring:* Due to the smaller ring strain present in 4-membered rings compared to 3-membered ones, these reactions possess sufficient thermodynamic driving force and hence constitute a viable strategy for oxetane synthesis. The 3-membered rings that are typically opened include epoxides and heteroatom-stabilised carbocations generated from alkenes (Scheme 10).

**Scheme 11:**
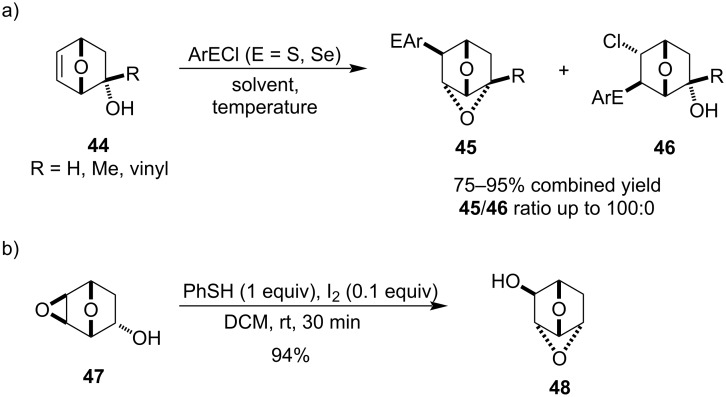
Synthesis of 4,7-dioxatricyclo[3.2.1.0^3,6^]octane skeletons.

In 1992, Arjona and co-workers examined the synthesis of 4,7-dioxatricyclo[3.2.1.0^3,6^]octane derivatives **45** via intramolecular cyclisation of the corresponding hydroxyalkene precursors **44** using arylsulphenyl and arylselenyl chlorides (Scheme 11a) [44]. The authors found that the secondary alcohol precursors were less reactive and that best results were obtained at low temperature (≤−50 °C) and in chlorinated solvents. The synthesis of these cages was later revisited by Le Drian et al. in 2011 who studied a Lewis acid-catalysed epoxide-opening cyclisation for the oxetane formation (Scheme 11b) [45]. The highest yield was obtained for the PhSH/I_2_ 10:1 activator under unusually mild conditions – the authors believed it is due to the oxa-bridge which facilitates the Lewis acid coordination.

**Scheme 12:**
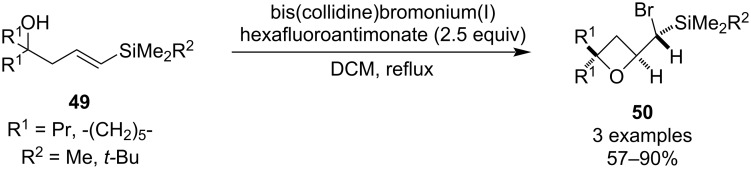
Silicon-directed electrophilic cyclisation of homoallylic alcohols.

In 2001, Rousseau and colleagues reported a preparation of oxetanes via silicon-directed electrophilic cyclisation of homoallylic alcohols **49** (Scheme 12) [46]. The reaction was promoted by a bromonium cation and moderate to high yields of oxetanes **50** were obtained. The authors claim the reaction was diastereospecific for disubstituted alkenes and high diastereocontrol (dr ≥ 80:20) was achieved for trisubstituted ones. Switching the bromonium activator to the iodonium analogue only led to a lower yield.

**Scheme 13:**
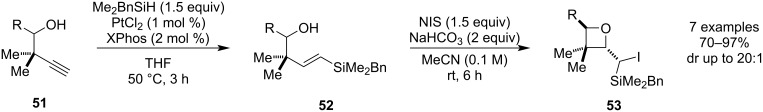
Hydrosilylation–iodocyclisation of homopropargylic alcohols.

This haloetherification method was further expanded by McLaughlin and Roberts in 2022 who reported a convenient two-step sequence towards tetrasubstituted oxetanes **53** via hydrosilylation of homopropargylic alcohols **51** followed by iodocyclisation (Scheme 13) [47], generating the 4-membered rings with excellent stereocontrol and in high yields. Optimisation studies of the second step revealed that the cyclisation must be carried out under mildly basic conditions to prevent possible side-reactions, specifically iododesilylation and oxetane opening by the relatively nucleophilic succinimide. Attempts to prepare the bulkier 2,3,3,4,4-pentasubstitued oxetanes were unsuccessful due to very poor conversions (<5%) and heating those reaction mixtures to increase the reaction rate only favoured the iododesilylation pathway.

**Scheme 14:**
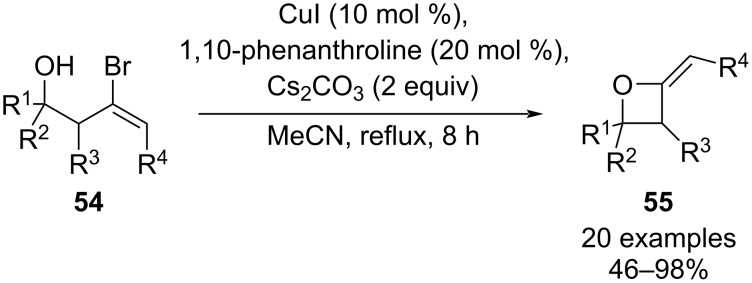
Cu-catalysed intramolecular *O*-vinylation of γ-bromohomoallylic alcohols.

**1.1.2 Cu-catalysed intramolecular cross-couplings:** In 2007, Li and Fang published a versatile Cu-catalysed intramolecular *O*-vinylation of γ-bromohomoallylic alcohols **54** yielding 2-alkylideneoxetanes **55** (Scheme 14) [48]. Good yields were obtained only when CuI was used in combination with 1,10-phenanthroline as a ligand, and this intramolecular Ullmann-type coupling was found to be compatible with primary, secondary and tertiary alcohols, with secondary alcohols exhibiting the following order of reactivity: aliphatic > allylic > benzylic. In case of trisubstituted C=C bonds, the cyclisation proceeded with retention of configuration, however, higher reaction temperatures were required due to the increased steric hindrance. Competition experiments revealed a preference for 4-*exo* ring closure over 5-*exo*, 6-*exo*, and 6-*endo* cyclization pathways – this may be attributed to precoordination of the Cu(I) catalyst to the alkoxide, which facilitates oxidative addition into the C–Br bond and results in the formation of a favorable five-membered Cu-containing intermediate [48].

**Scheme 15:**
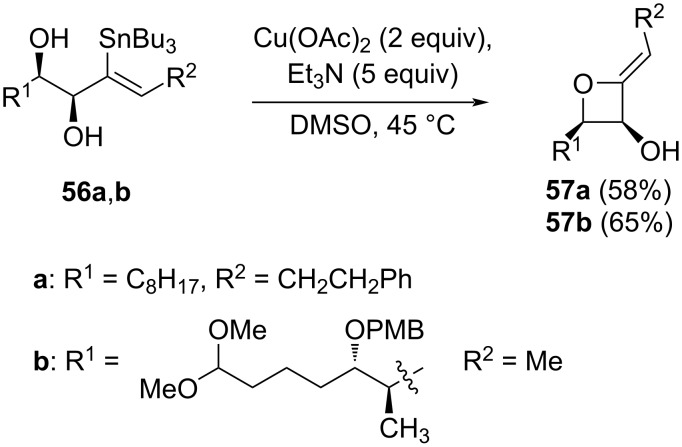
Cu-catalysed intramolecular cross-coupling of hydroxyvinylstannanes.

Ten years later, while developing the transformation of alkenylstannanes into acetylated acyloins, Fürstner et al. [49] reported the synthesis of two 2-alkylideneoxetanes **57** via an unexpected Cu-catalysed intramolecular cross-coupling of hydroxyvinylstannanes **56** (Scheme 15). Besides the good yields, the stannane substrates were also readily prepared by *trans*-hydrostannation [50] of the corresponding alkyne precursors, so this report potentially introduced a new method for oxetane synthesis through an intramolecular Chan–Lam-type coupling.

Due to the high reactivity of the exocyclic enol ether induced by the ring strain, 2-alkylidene-, particularly 2-methylideneoxetanes have become valuable intermediates in oxetane chemistry as they readily undergo a variety of chemical transformations, including ring-opening reactions with nucleophiles, epoxidations, cyclopropanations and [3 + 2] cycloadditions with nitrile oxides [51–52]. In addition, Howell et al. recently disclosed a general method for the synthesis of 2-halomethylideneoxetanes through halogenation of 2-methylideneoxetanes using *N*-halosuccinimides, and illustrated their synthetic utility by Suzuki–Miyaura, Sonogashira and Buchwald–Hartwig coupling reactions [53].

In addition to Cu-mediated intramolecular cross-couplings, 2-alkylideneoxetanes can also be accessed by formal [2 + 2] cycloadditions, which are discussed in chapter 1.3.2.

#### 1.2 C–C Bond-forming cyclisations

**Scheme 16:**
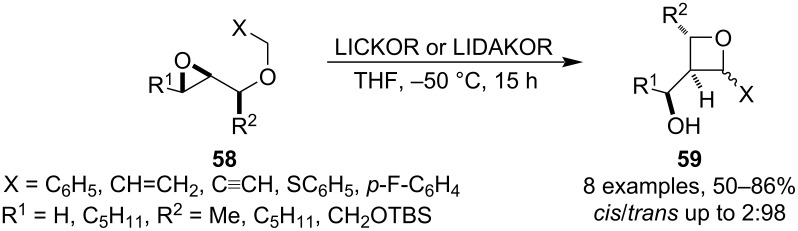
Isomerisation of oxiranyl ethers containing weakly carbanion-stabilising groups.

This relatively uncommon strategy is usually based on an ionic mechanism in which an S_N_2 substitution takes place after deprotonation of a suitably functionalised ether at the α-carbon. Therefore, a stabilising group must be incorporated to control regioselectivity of the deprotonation as well as to increase the acidity of the α-hydrogen. Mordini et al. showed that even weakly stabilising groups such as phenyl, vinyl, ethynyl or sulphide are sufficient if a superbase such as LIDAKOR or LICKOR is used (Scheme 16) [54–55]. The reaction tends to be remarkably regioselective (in terms of the epoxide opening) and stereoselective, however, it should be treated with caution in case of allyl ethers as they can also react through the terminal carbon and deliver tetrahydrooxepines, especially, if the oxirane is monosubstituted (**58**, R^1^ = H).

**Scheme 17:**
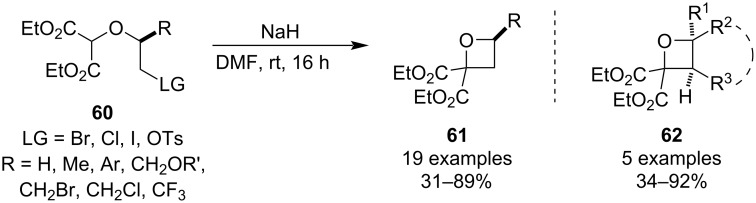
Cyclisation of diethyl haloalkoxymalonates.

The deprotonation step can also be enabled by employing classical electron-withdrawing groups such as esters, and a particularly powerful methodology was disclosed by Bull and Davis in 2014 [56]. The combination of the malonate functionality with halides (mostly bromine) as the leaving group allowed for much milder and more convenient cyclisation conditions, delivering trisubstituted oxetanes **61** in high yields (Scheme 17). The authors also developed a practical synthesis of the precursor **60** from diazomalonate based on a Rh-catalysed O–H insertion, and further applied this cyclisation methodology to a preparation of tetrasubstituted oxetanes **62**, including bicyclic analogues.

**Scheme 18:**
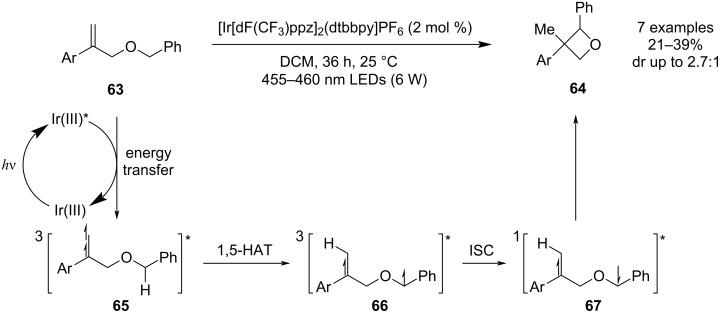
Synthesis of oxetanes through a 1,5-HAT/radical recombination sequence.

In 2024, Liu, Shi, Wei and co-workers published the first radical cyclisation of ethers leading to polysubstituted oxetanes **64** (Scheme 18) [57]. The mechanism is based on a 1,5-HAT/radical recombination sequence where the H-atom transfer is triggered by an S_0_ → T_1_ excitation of the starting allyl ether **63** using an iridium photosensitiser and blue light for irradiation. The method employs mild reaction conditions and exhibits excellent functional group tolerance (demonstrated by synthesising a large library of azetidine analogues), but tends to deliver the oxetane products in rather low yields due to fragmentation of the biradical intermediate **67** through a Norrish-type II process.

#### 1.3 [2 + 2] Cycloadditions

**Scheme 19:**
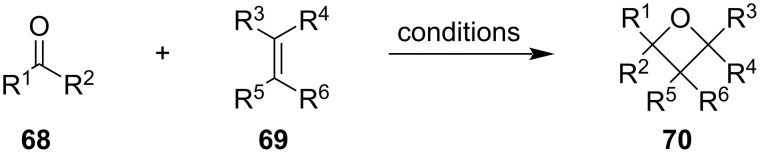
General approach to oxetanes via [2 + 2] cycloadditions.

Another widely used method for oxetane synthesis is the [2 + 2] cycloaddition between carbonyls and olefins (Scheme 19), and the two main variations include light-induced Paternò–Büchi reactions and Lewis acid- or base-catalysed formal [2 + 2] cycloadditions. The main advantages of these reactions are their great versatility and atom economy.

**1.3.1 Photocycloadditions:** The first example of a [2 + 2] photocycloaddition was observed by Paternò in 1909 who reported oxetane formations from mixtures of tri- and tetrasubstituted alkenes with carbonyls exposed to sunlight [58]. More than 40 years later, Büchi et al. decided to reinvestigate this novel reaction in order to develop a practical laboratory procedure for synthesising polysubstituted oxetanes [59]. They reported a synthesis of three different oxetanes by irradiating mixtures of 2-methyl-2-butene with benzaldehyde, acetophenone and butyraldehyde in a mercury vapour illuminator and since then, the reaction has become known as the Paternò–Büchi reaction. Throughout the years after the report, investigations in this field were focused on identifying new reactive alkene and carbonyl components, developing photosensitisers that would allow performing the reaction under visible light irradiation, or coupling the reaction to other light-induced processes to produce new classes of products.

**Scheme 20:**
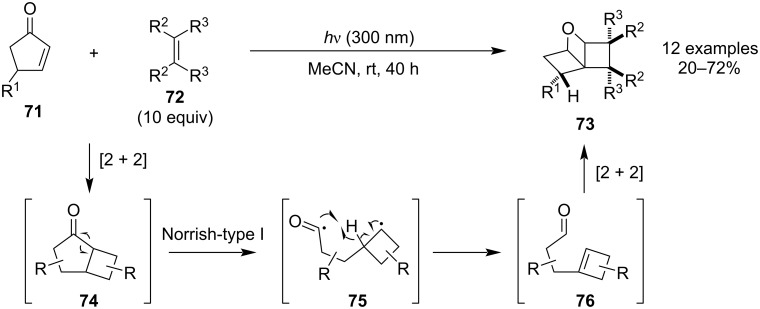
Synthesis of tricyclic 4:4:4 oxetanes through a photochemical triple cascade reaction.

In 2018, Aitken and co-workers reported a synthesis of previously unknown tricyclic 4:4:4 oxetanes **73** through a photochemical triple cascade reaction starting from simple cyclopentenones **71** and symmetric alkenes **72** (Scheme 20) [60]. Although the reaction is rather low-yielding (mostly below 30%), it tends to give high diastereoselectivities. The mechanism is believed to proceed through the following steps: [2 + 2] photocycloaddition, Norrish-type I cleavage, γ-H transfer and Paternò–Büchi reaction.

**Scheme 21:**
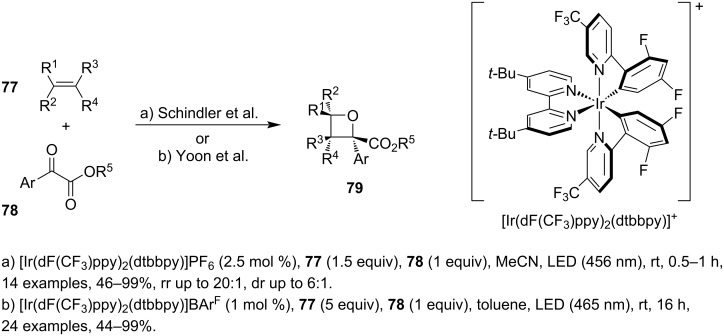
Iridium-catalysed Paternò–Büchi reaction between α-ketoesters and simple alkenes.

In 2020, two highly similar methodologies based on a visible-light-mediated Paternò–Büchi reaction between simple alkenes **77** and α-ketoesters **78** were reported independently and shortly after one another (Scheme 21) [61–62]. They both use blue light for irradiation and employ the same cationic iridium photosensitiser, but they differ in solvent, catalyst loading and power of the light source which is reflected mainly in the reaction times. The cycloadducts were obtained in high yields and both electron-rich and poor aryls were tolerated, as well as potentially reactive functional groups such as alkenes, alkynes, halides or azides.

**Scheme 22:**
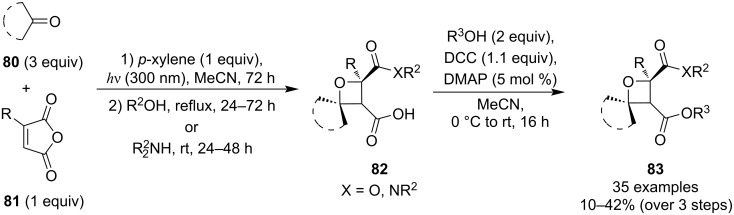
Three-step synthesis of spirocyclic oxetanes **83** via Paternò–Büchi reaction, nucleophilic ring opening and DCC-mediated esterification.

In 2023, Coote et al. developed a synthetic approach to functionalised spirocyclic oxetanes **83** by combining a Paternò–Büchi reaction with succinic anhydride opening and esterification (Scheme 22) [63]. The key intermediates **82** produced after the first two steps were obtained as single isomers and in moderate yields after a single chromatographic purification, thus making the overall synthetic sequence relatively efficient. Also, thanks to the addition of 1 equivalent of *p*-xylene, which is claimed to suppress alkene [2 + 2] dimerisation, this methodology enables performing photocycloadditions between electron-deficient alkenes and aliphatic ketones which had been usually avoided.

**Scheme 23:**
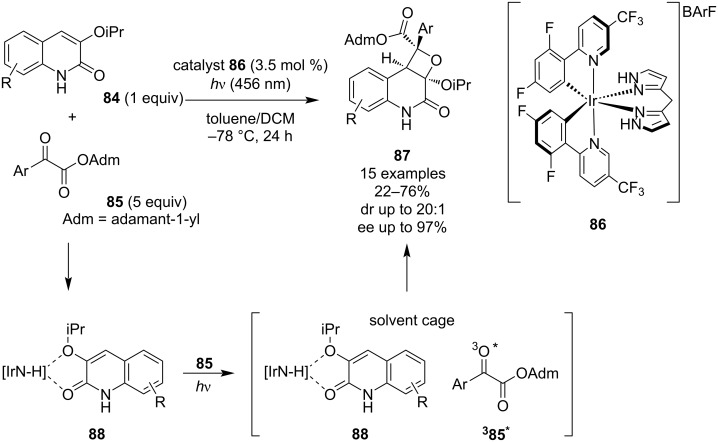
Enantioselective Paternò–Büchi reaction catalysed by a chiral iridium photocatalyst.

One year later, Yoon and co-workers published the first highly enantioselective Paternò–Büchi reaction between quinolones **84** and ketoesters **85** (Scheme 23) based on the rebound triplet mechanism [64]. The stereochemical behaviour of the reaction is controlled by a novel hydrogen-bonding chiral iridium photocatalyst **86**, delivering oxetane products **87** in excellent enantiomeric excess. In this unique photocycloaddition mechanism, previously described by the same research group [65–66], the Ir catalyst initially interacts through hydrogen bonds with the quinolone substrate **84**, and then upon irradiation, energy transfer occurs to the unbound ketone which enantioselectively reacts with the bound quinolone. Unfortunately, investigations of the substrate scope revealed that the reaction stereoselectivity is highly dependent on the structure of the ketoester component and tends to be unpredictable.

**Scheme 24:**
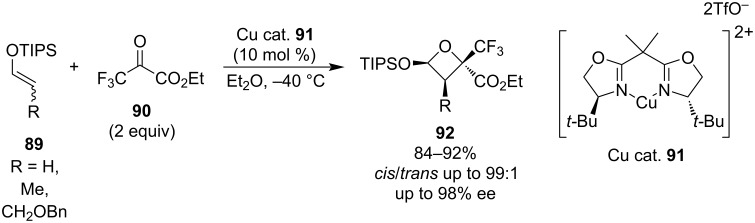
Synthesis of polysubstituted oxetanes **92** via Cu(II)-mediated formal [2 + 2] cycloadditions.

**1.3.2 Formal cycloadditions:** These stepwise processes typically take place between an enolate or enol ether and a carbonyl under Lewis acid or base catalysis and proceed through a double addition mechanism. In 2011, Mikami et al. developed a catalytic asymmetric oxetane synthesis from silyl enol ethers **89** and trifluoropyruvate **90** using a chiral Cu(II) complex (Scheme 24) [67]. Besides excellent yields, they also observed very high *cis*/*trans* ratios and enantioselectivities.

**Scheme 25:**
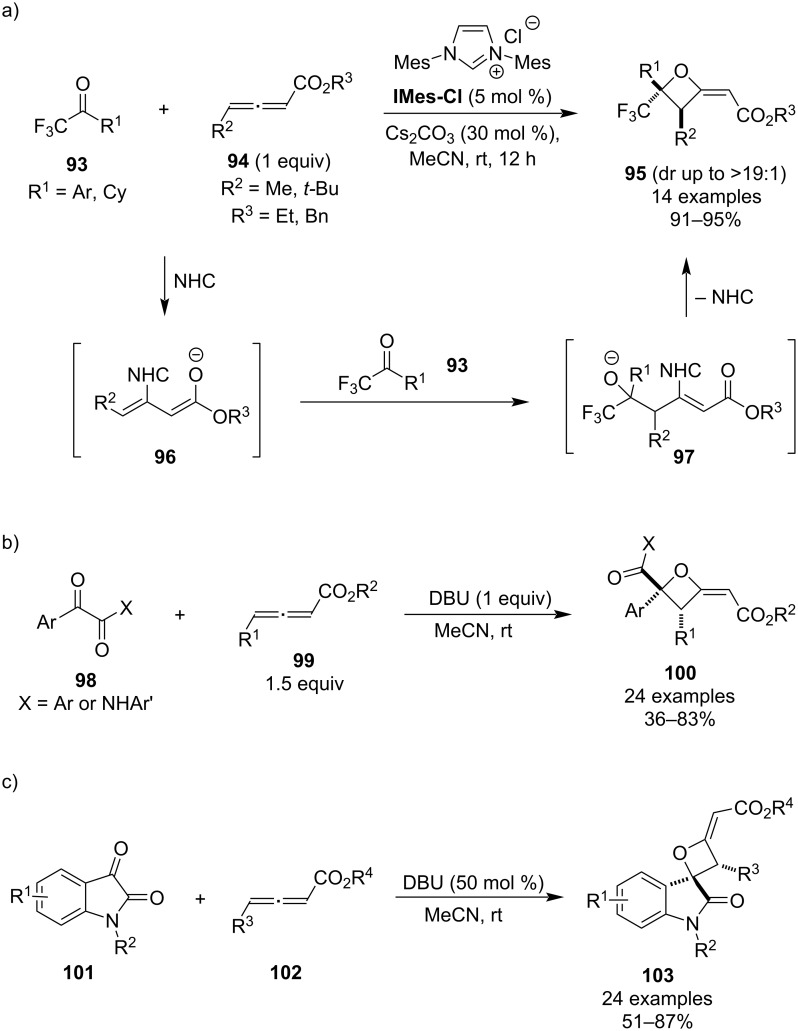
Synthesis of alkylideneoxetanes via NHC- and DBU-mediated formal [2 + 2] cycloadditions.

In 2018, Scheidt and colleagues disclosed the first *N*-heterocyclic carbene (NHC)-catalysed [2 + 2] annulation between trifluoromethyl ketones and γ-substituted allenoates (Scheme 25a) [68]. The resulting 2-alkylideneoxetane products **95** were generally obtained in >90% yields and good diastereomeric ratios favouring the *trans*-isomer, however, a reversed diastereoselectivity was observed for *ortho*-substituted aryls due to steric factors. The investigation of the substrate scope revealed that while the reaction shuts down in the absence of the trifluoromethyl group in the ketone component, it still runs smoothly, if the aryl is substituted for cyclohexyl. The proposed mechanism, supported by control experiments, deuterium exchange studies and energy calculations, consists of the following steps: conjugate addition of the carbene to the allenoate, regioselective addition of the resulting extended enolate **96** to the ketone, cyclisation via addition/elimination and base-catalysed epimerisation towards the thermodynamically more stable diastereomer. In 2019, Nair and co-workers showed that this formal cycloaddition can also be performed with 1,2-dicarbonyls as electrophiles and under an amidine base catalysis (Scheme 25b) [69]. Although this methodology employs equimolar catalyst loadings, the products **100** are obtained with complete diastereoselectivity and mostly in moderate to good yields. As for the mechanism, it is assumed to be analogous to the NHC-variant. Four years later, Somappa and colleagues further extended the scope of this methodology to isatins as the ketone substrates, generating the corresponding spiro-oxetanes **103** in similarly good yields and, just like before, as single diastereomers (Scheme 25c) [70].

#### 1.4 Ring expansions

**Scheme 26:**
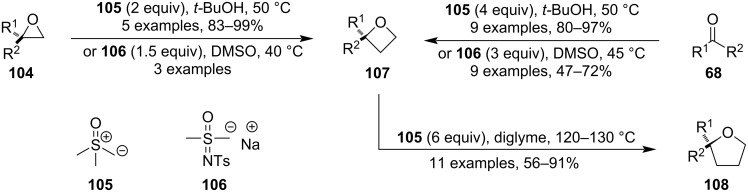
Use of sulphur-stabilised carbanions in ring expansions.

This strategy allows for a facile synthesis of oxetanes from epoxides using sulphur-stabilised carbanions such as dimethyloxosulphonium methilide (**105**) or *S*-methyl-*S*-(sodiomethyl)-*N*-(4-tolylsulphonyl)sulphoximine (**106**) (Scheme 26) [71–72]. Because these reagents are also known to induce epoxidation when reacted with a ketone or aldehyde (Corey–Chaykovsky reaction), oxetanes can also be conveniently prepared from carbonyl compounds using a larger excess (>2 equiv) of the carbanion. However, harsh reaction conditions should be avoided as using 6 equiv of the ylide **105** with respect to the oxetane and elevated temperatures (120–130 °C) can lead to another ring expansion producing tetrahydrofurans **108** [73].

**Scheme 27:**
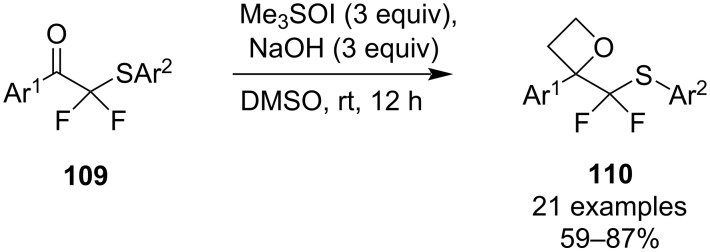
Synthesis of α,α-difluoro(arylthio)methyl oxetanes.

In 2022, Zhu et al. employed this strategy in their synthesis of α,α-difluoro(arylthio)methyloxetanes **110** using an excess of the sulphoxonium ylide under very mild conditions (Scheme 27), most likely enabled by the high electrophilicity of the carbonyl [74]. The oxetane products were obtained in good to high yields and further transformed into useful sulphone, butenolide and tetrahydrofuran derivatives.

**Scheme 28:**
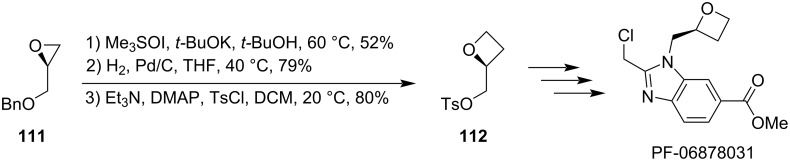
Ring expansion in an industrial synthesis of PF-06878031.

Besides laboratory-scale reactions, this ring expansion has also found applications in industry, specifically for a multi-kilogram synthesis of PF-06878031 which is a key structural fragment of various GLP-1 receptor agonists [75]. As depicted in Scheme 28, the treatment of enantiomerically pure *O*-benzylglycidol (**111**) with ylide **105** generated from Me_3_SOI followed by hydrogenolysis and sulphonylation delivered oxetane tosylate **112** in 33% overall yield, which was then used in a 4-step sequence to produce PF-06878031 in >99% purity and a combined yield of 1,500 kg across multiple facilities.

#### 1.5 Ring contractions

**Scheme 29:**
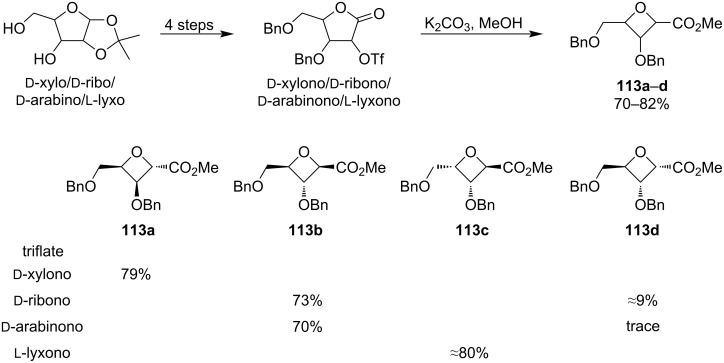
Ring contraction of triflated 2-hydroxy-γ-lactones.

Another strategy for oxetane synthesis is a ring contraction of 5-membered rings. There are two main approaches: the first one comprises an alcoholysis of γ-lactones containing a leaving group (typically a triflate) at C-2 followed by spontaneous Williamson etherification. This method was extensively studied by Fleet and Jenkinson [76] and was applied for example for the synthesis of oxetane carboxylic esters **113a**–**d**, which are valuable precursors of oxetane nucleosides (Scheme 29) [77]. The triflates were prepared from the corresponding 1,2-*O*-isopropylidenepentofuranose sugars in 4 steps and then treatment with methanolic K_2_CO_3_ smoothly induced the ring contraction, affording the oxetane products in 70–82% yield. Interestingly, only the xylono-lactone showed complete inversion of the configuration at C-2 while the lyxono-lactone showed complete retention of the configuration. The ribono- and arabinono-lactone displayed predominant retention and inversion, respectively. Switching the triflyl group for the less reactive mesyl almost completely shut down the ring contraction.

**Scheme 30:**
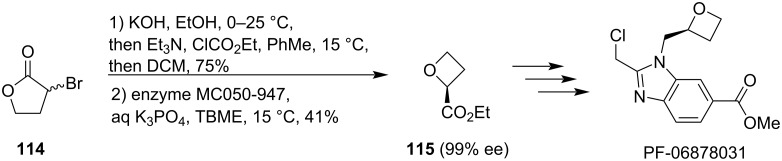
Ring contraction in an industrial synthesis of PF-06878031.

In 2024, Twiddle et al. showed that this ring contraction can also be effected from lactones containing bromine as the leaving group and used this approach to develop a 2nd generation industrial synthesis of PF-06878031 (Scheme 30) [78]. Initially, racemic 2-bromo-γ-butyrolactone (**114**) was contracted by ethanolic KOH into oxetanecarboxylic acid, which was then esterified and the enantiomeric ethyl esters were resolved by an enzymatic hydrolysis. Finally, the enantioenriched ester **115** was successfully employed in several multi-kilogram syntheses to generate >1,500 kg of >99% pure PF-06878031.

**Scheme 31:**
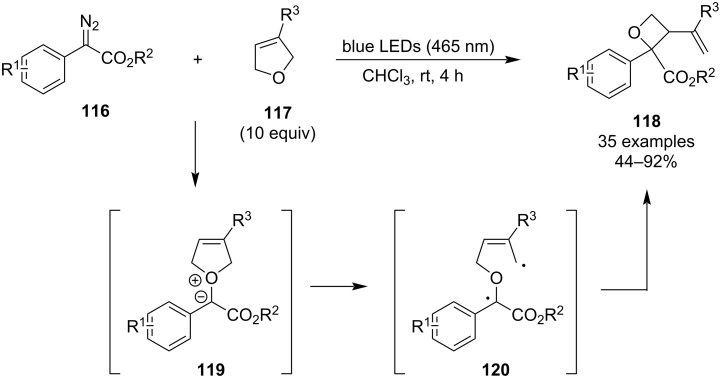
Photochemical ring contraction of 2,5-dihydrofurans by aryldiazoacetic acid esters.

The second method was developed only recently by Xia and co-workers who in 2022 disclosed a versatile photochemical ring contraction of 2,5-dihydrofurans **117** enabled by aryldiazoacetic acid esters **116** (Scheme 31) [79]. The reaction is promoted by visible light at room temperature, requires no protecting atmosphere or additives, and the resulting polysubstituted oxetanes are obtained in moderate to good yields. Additional advantages include scalability and high chemoselectivity as various functional groups were tolerated, including halogens, nitriles, alkenes and heteroaryls. On the other hand, this methodology suffers from relatively low diastereoselectivity as the dr lies between 1:1 and 2:1. DFT calculations suggested the reaction proceeds through nitrogen elimination, oxonium ylide **119** formation, homolytic cleavage and radical recombination.

#### 1.6 O–H insertions

**Scheme 32:**
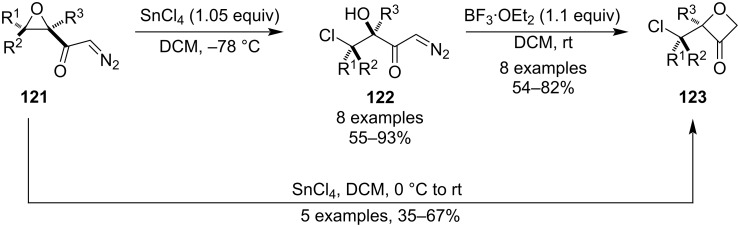
Synthesis of 3-oxetanones via O-H insertion of carbenes.

This strategy is relatively uncommon and it relies on the insertion of carbenes into O–H bonds. In 1992, Zwanenburg et al. published an efficient synthesis of 3-oxetanones **123** from α,β-epoxydiazomethyl ketones **121** (Scheme 32) [80]. First, the epoxides were regioselectively opened by SnCl_4_ at −78 °C in a *syn* manner to give the corresponding chlorohydrins **122**, which then upon treatment with BF_3_·OEt_2_ underwent the O–H insertion producing oxetanones **123** in good yields. The authors also found that increasing the temperature to rt during the epoxide opening afforded the oxetanones directly in a single step.

**Scheme 33:**
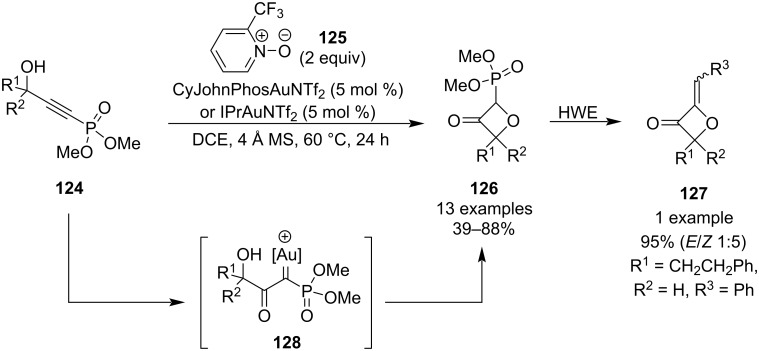
Synthesis of phosphonate oxetanones via gold-mediated alkyne oxidation/O–H insertion.

In 2022, Hashmi and colleagues developed a practical approach towards 2-alkylideneoxetan-3-ones **127** based on a convenient one-step synthesis of phosphonate-substituted 3-oxetanones **126** which readily undergo the Horner–Wadsworth–Emmons (HWE) reaction (Scheme 33) [81]. These oxetanone precursors are generated from easily accessible alkynylphosphonates **124** via gold-mediated alkyne oxidation by pyridine *N*-oxide **125** followed by a formal O–H-insertion of the resulting metallacarbene intermediate **128**. The optimised procedure requires 5 mol % loadings of the gold catalyst, anhydrous conditions and mild heating to afford the products in moderate to good yields. The reaction worked well for both aliphatic and aryl-substituted substrates but best results were obtained for shorter hydrocarbon side chains and 2,6-disubstituted phenyls. The authors demonstrated the applicability of the phosphonates **126** as HWE reagents in one example.

### 2 Derivatisation of 3-oxetanone

**Scheme 34:**
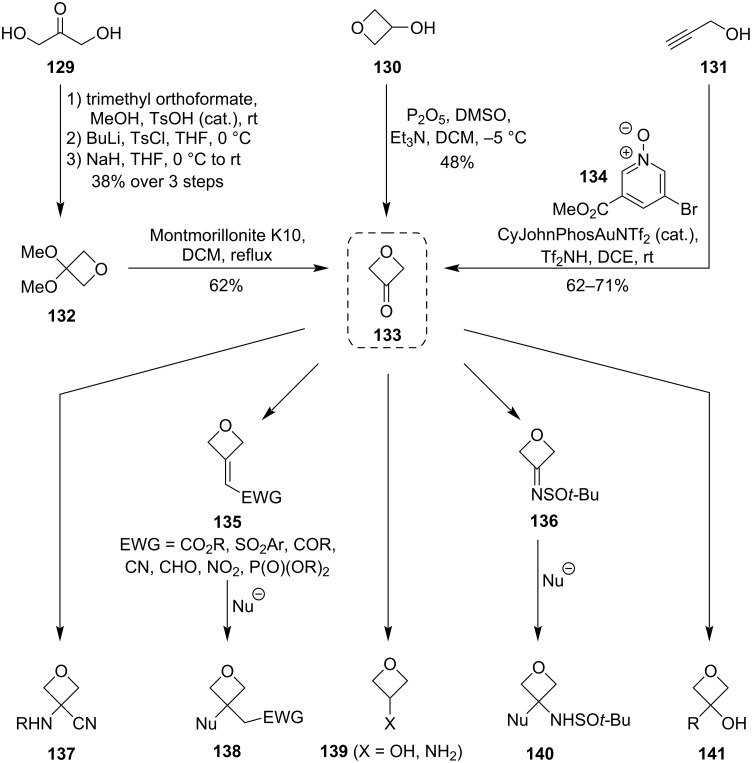
Syntheses and common derivatisations of 3-oxetanone.

Because of the highly beneficial properties of oxetanes as isosteric replacements, there is a strong need for a convenient access to 3-substituted oxetanes. Besides constructing the 4-membered ring de novo, another approach to these derivatives can proceed through functionalisations of readily available oxetane-containing building blocks. The most versatile precursor of these building blocks is 3-oxetanone (**133**) which was first recognised by Carreira and colleagues (Scheme 34). Besides describing its possible applications, the research group has also developed two practical and scalable syntheses of this compound: one based on oxidation of 3-oxetanol (**130**) (obtained from epichlorohydrin), and the other on a Williamson etherification of dihydroxyacetone (**129**) [10–12]. In 2010, Zhang et al. added another synthetic route to the repertoire which proceeds through a gold-catalysed oxidation of propargyl alcohol (**131**) [82]. Scheme 34 also shows selected transformations from the publications by Carreira et al. to provide a brief insight into what oxetane building blocks can be prepared from 3-oxetanone: these include the products **137** through Strecker reactions, aldol-type condensations and phosphorus ylide-based olefinations (**135**) with subsequent Michael additions (**138**), or sulphinimine and ketone additions (**140** and **141**, respectively). These common functionalisations are also well documented in a recent comprehensive work by Volochnyuk and Ryabukhin et al. who analysed the oxetane core tolerance towards various reaction conditions such as oxidations, reductions, alkylations or C–C bond formations [83]. Since the general recognition of 3-oxetanone as the principal building block, a large number of new derivatisation methods have been developed – these expand not only the scope of synthetic strategies towards known oxetane building blocks, but also the library of medicinally relevant oxetane derivatives. This chapter summarises the recent functionalisations of 3-substituted oxetanes whose synthesis can be traced back to 3-oxetanone.

**Scheme 35:**
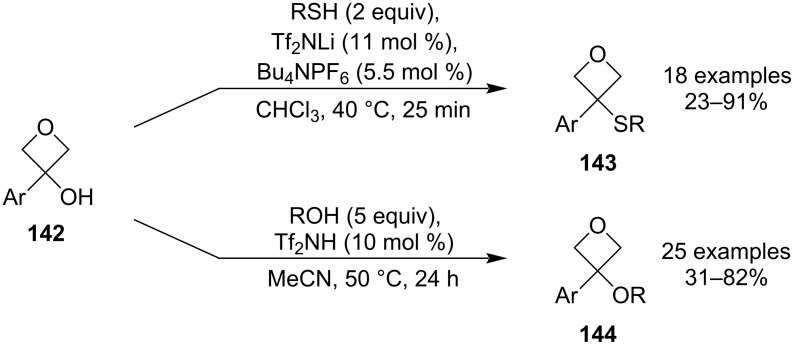
S_N_1 substitution of 3-aryloxetan-3-ols by thiols and alcohols.

In 2018, Bull and co-workers disclosed the first lithium-catalysed thiol alkylation using electron-rich 3-aryloxetan-3-ols **142** (Scheme 35) [84]. This protocol is completely chemoselective as no ring-opening was observed and the resulting oxetane sulphides **143** were obtained in high yields. Further transformations were also investigated such as a Suzuki coupling (at the aryl group) or oxidation to sulphoxides and sulphones, demonstrating the versatility of these products. In addition, their medicinally relevant physicochemical properties such as lipophilicity, clearance and cell membrane permeability were measured and the results suggested that these substrates might be promising bioisosteric replacements for thioesters. Five years later, the research group expanded the scope of this methodology to include alcohols as the nucleophiles (Scheme 35) [85], thus avoiding the previous need for strong base-induced alkylations with alkyl halides. The protocol is similarly mild, employs a Brønsted acid catalyst and affords the ether products **144** in moderate to high yields.

**Scheme 36:**
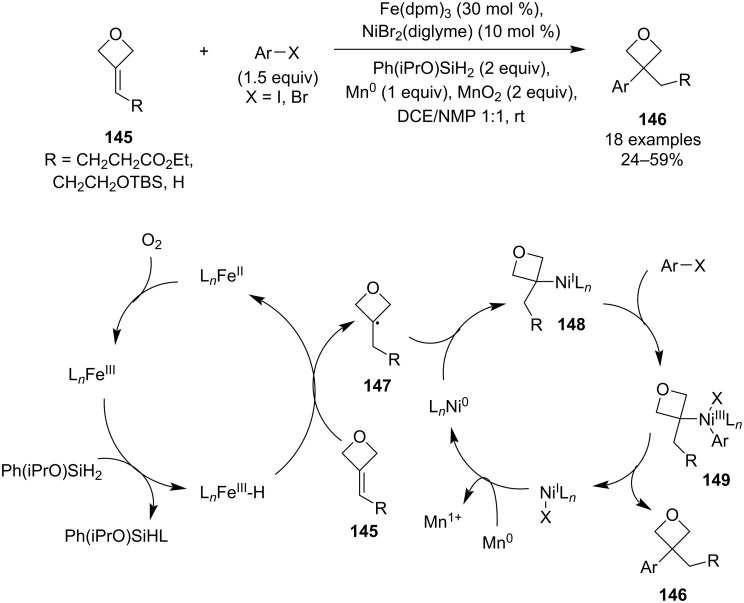
Fe–Ni dual-catalytic olefin hydroarylation towards 3-alkyl-3-(hetero)aryloxetanes.

In 2018, Shenvi and colleagues reported a Markovnikov-selective olefin hydroarylation based on an unprecedented Fe–Ni dual catalysis (Scheme 36) [86], which constitutes a more versatile alternative to Minisci or Friedel–Crafts alkylations. The reaction couples mono-, di- and trisubstituted olefins with (hetero)aryl halides, and it was used to prepare a relatively large library of 3-alkyl-3-(hetero)aryloxetanes **146** in moderate yields. Investigation of the reaction scope identified electron-rich, -neutral and -poor arenes as well as vinyl bromides as viable coupling partners, and the authors also applied this methodology for a novel, simplified synthesis of two medicinally relevant oxetane precursors. The mechanism was proposed to start with formation of a Fe(III)–H species which delivers a hydrogen radical to the less-substituted end of the alkene. The resulting tertiary C-centred radical **147** then couples with a Ni(0) complex (generated by a reduction of the Ni(II) pre-catalyst) and after an oxidative addition of the aryl halide, the disubstituted oxetane product is generated by a reductive elimination. Finally, the two catalytic cycles are closed by an oxidation/reduction process: the Fe(II) species is reoxidised by atmospheric oxygen and the Ni(I) complex is reduced by the added manganese powder.

**Scheme 37:**
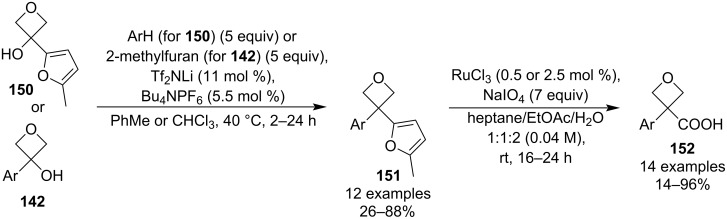
Synthesis of 3-aryloxetan-3-carboxylic acids.

In 2020, Bull et al. published a short synthesis of 3-aryloxetan-3-carboxylic acids **152** employing a Friedel–Crafts alkylation (which builds on their previous alkylation of phenols [87]) and a selective furan oxidative cleavage (Scheme 37) [88]. The oxidation protocol uses a catalytic amount of a high oxidation state ruthenium, allowing for a facile purification (simple acidic/basic workup) and is very mild, scalable and high-yielding. However, the yield tends to get rather low (below 25%), if the oxetane bears a heterocycle (e.g., indole or thiophene), most likely due to competing oxidations of the heteroarenes.

**Scheme 38:**
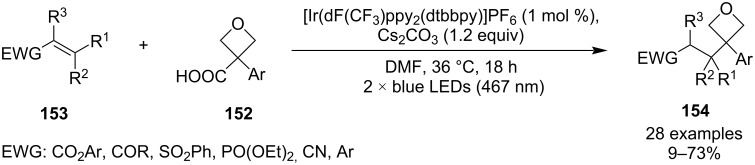
Decarboxylative alkylation of 3-aryloxetan-3-carboxylic acids.

This practical synthesis of 3-aryloxetan-3-carboxylic acids potentially opened the door to installation of a wide range of substituents into the 3-position through a decarboxylative radical coupling, which was eventually exploited by Duarte and Bull et al. in 2023 (Scheme 38) [89]. The decarboxylation was induced by a photochemical oxidation using an iridium catalyst, and the resulting benzylic radicals were coupled with activated alkenes through a Giese addition which was irreversible due to the strained nature of the starting radicals. As a result, radical dimerization was minimal and the 3,3-disubstituted oxetane products were delivered in moderate yields. In addition, the method uses low catalyst loadings, tolerates various functional groups (e.g., esters, ketones, nitriles, phosphonates) and seems to be insensitive to common deviations from the optimised conditions.

**Scheme 39:**
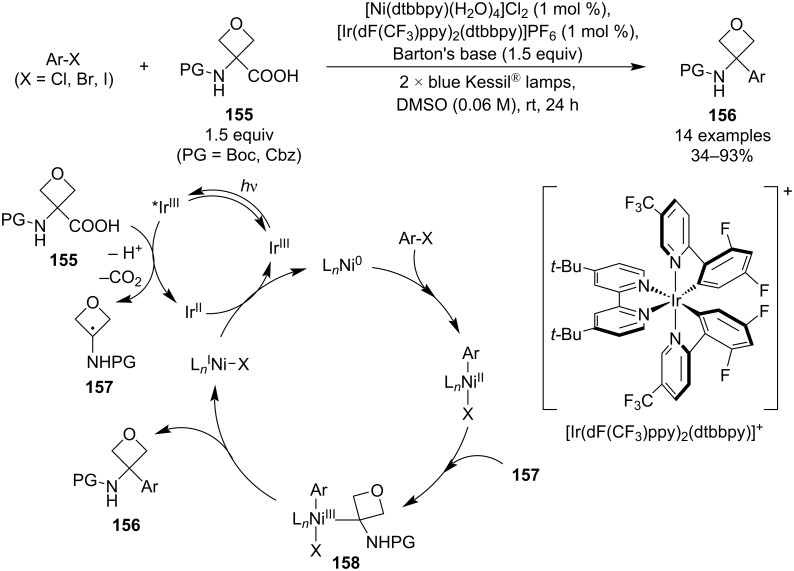
Synthesis of 3-amino-3-aryloxetanes via photoredox/nickel cross-coupling catalysis.

In 2020, Terrett, Huestis and co-workers reported an unprecedented synthesis of 3-aryl-3-aminooxetanes **156** from amino acids **155** utilising a combination of photoredox and nickel cross-coupling catalysis (Scheme 39) [90]. The reaction uses low catalyst loadings, gives moderate to excellent yields and tolerates various functional groups including esters, ketones, sulphones and heteroaryls. The mechanistic proposal, supported by DFT calculations, starts with an oxidative decarboxylation to give an aminooxetanyl radical **157**. This species is in turn coupled with the aryl halide by the active Ni(0) catalyst (generated in situ by reduction of the Ni(II) pre-catalyst) via oxidative addition, radical coupling and reductive elimination. The last step is a single-electron transfer between the resulting Ir(II) and Ni(I) complexes, regenerating the active catalysts and closing the two cycles.

**Scheme 40:**
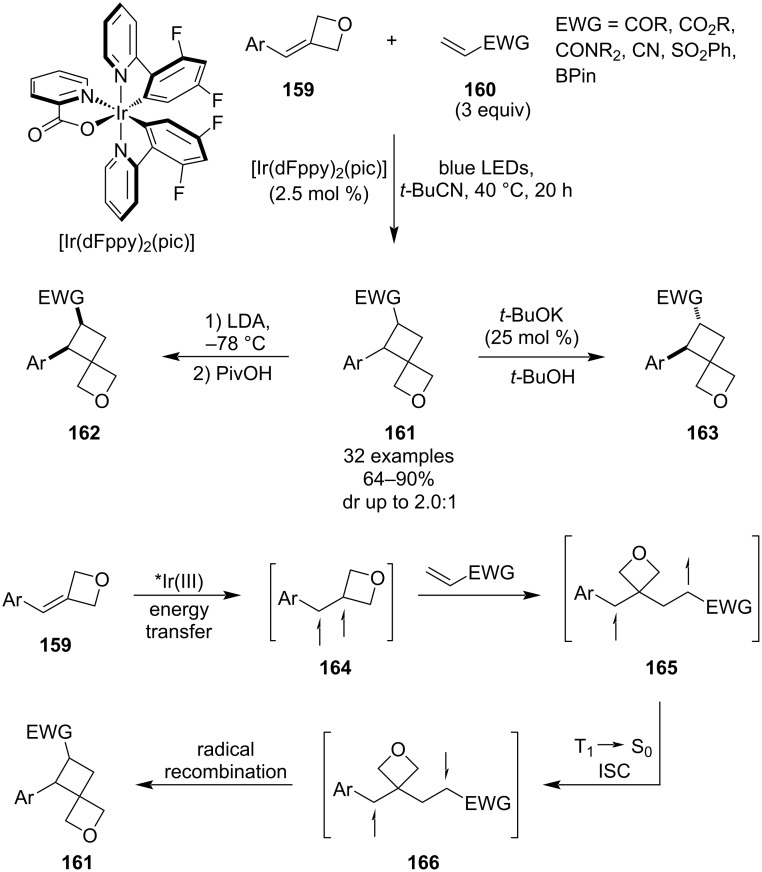
Intermolecular cross-selective [2 + 2] photocycloaddition towards spirooxetanes.

In 2021, Romanov-Michailidis and Knowles et al. published a light-induced cross-selective [2 + 2] cycloaddition between 3-(arylmethylidene)oxetanes **159** and electron-deficient alkenes **160** (Scheme 40) [91]. The methodology used a commercially available iridium-based photosensitiser and blue-light irradiation at a slightly elevated temperature. The resulting 2-oxaspiro[3.3]heptanes **161** were obtained in high yields for a broad scope of aryls (substituted phenyls, 5-membered and fused bicyclic heteroaryls) as well as electron-withdrawing groups including esters, amides, nitriles or sulphones. The authors also developed conditions for the product epimerisation to obtain diastereoenriched spirocycles, utilising *t*-BuOK/*t*-BuOH for the *anti*-diastereomer **163** and LDA/PivOH at −78 °C for the *syn*-diastereomer **162** (dr >10:1 in both protocols). Mechanistic and computational studies suggested the following series of steps: excitation of **159** via energy transfer from the photoexcited Ir complex, Giese-type addition of the resulting triplet diradical **164** to the electron-deficient alkene, intersystem crossing generating a singlet diradical **166** and intramolecular radical recombination.

**Scheme 41:**
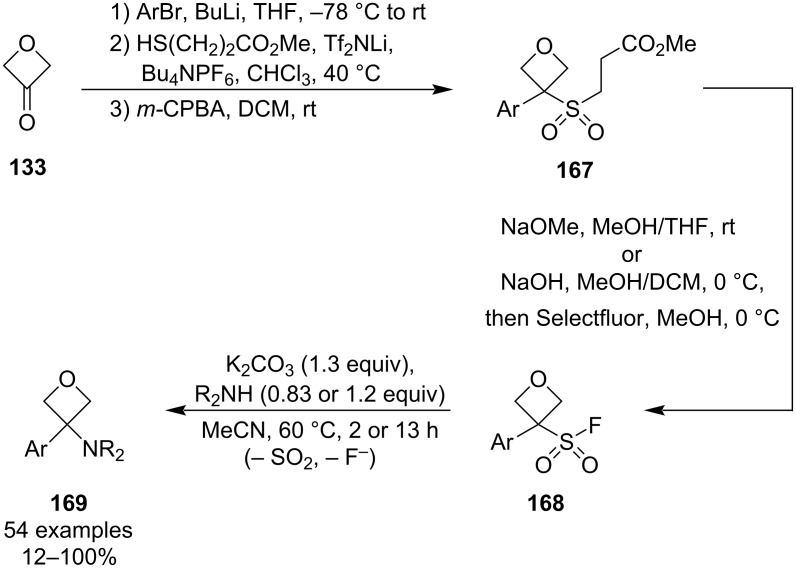
Synthesis of 3-aryl-3-aminooxetanes via defluorosulphonylative coupling.

In 2022, Bull and colleagues disclosed an unprecedented synthesis of 3-aryl-3-aminooxetanes **169** through a defluorosulphonylative coupling of sulphonyl fluorides **168** (Scheme 41) [92]. Because this novel methodology mimics the classical amide coupling strategy, it allows for a direct use of the established amine libraries and thus provides a rapid access to benzamide bioisosteres. The oxetane sulphonyl fluorides can be conveniently prepared in 4 steps from 3-oxetanone and are sufficiently stable at room temperature and to air/moisture. The coupling proceeds upon gentle heating in the presence of K_2_CO_3_ as the only additive and affords the aminooxetanes in high yields, even for challenging amines. The combination of simple reaction conditions and excellent chemoselectivity makes this protocol very robust and suitable for both the academia and industry. Kinetic and computational experiments support an S_N_1 mechanism via loss of sulphur dioxide and oxetane carbocation formation. Two years later, the group extended the scope of nucleophiles to azoles, sulphoximines (and other HN=S species), phosphites, phosphonites or secondary phosphine oxides, and developed selective reaction conditions for the alternative sulphur–fluoride exchange (SuFEx) pathway by employing hard anionic nucleophiles, thus providing access to oxetane sulphonamides, sulphonyl azides, sulphonates and sulphones [93].

**Scheme 42:**
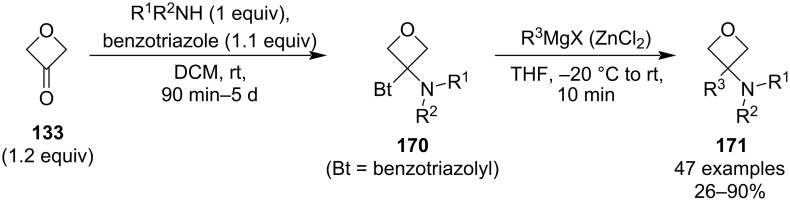
Two-step synthesis of amide bioisosteres via benzotriazolyl Mannich adducts **170**.

In 2024, Soós et al. published a similarly mild protocol for the synthesis of amide bioisosteres which utilises Katritzky’s benzotriazole chemistry (Scheme 42) [94]. Unlike the Bull’s methodology, the synthesis starts with the preparation of a reactive amine species **170** which is then reacted with a Grignard or, in case of ester-containing substrates, with an organozinc reagent. Because this method involves only two simple steps and is not limited to aryl groups, it provides a more rapid access to a potentially even larger library of amide isosteres. The scope of the carbon nucleophiles is very broad and includes alkyls, alkenyls, alkynyls, aryls and heteroaryls (e.g., pyridine, indole, thiophene), as well as (poly)substituted phenyls bearing a nitrile or halogen(s). On the other hand, the benzotriazole-adduct formation proceeds poorly with sterically demanding secondary amines and fails with primary amines. However, the latter may be overcome by adding a temporary benzyl group on the amine which can be subsequently cleaved by hydrogenolysis as demonstrated by the authors. Finally, it was shown that this synthesis is easily scalable up to a 100 g scale.

**Scheme 43:**
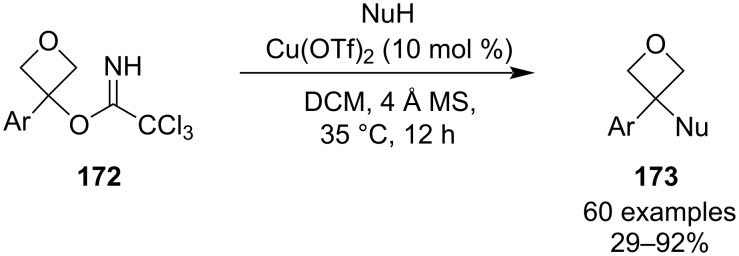
Functionalisation of oxetanyl trichloroacetimidates **172**.

In the same year, Zhang et al. developed another highly modular route to 3,3-disubstituted oxetanes **173** via derivatization of oxetanyl trichloroacetimidates (Scheme 43) [95]. This method, inspired by a Schmidt glycosylation, provides easy access to a large library of functionalised oxetanes in moderate to excellent yields and under very simple reaction conditions, which are compatible with a wide range of nucleophiles including alcohols (primary, secondary and tertiary), phenols, aliphatic and aromatic amines or heteroaryls (e.g., furan and indole). A Hammett analysis and control experiments showed that the reaction proceeds via an S_N_1 mechanism and that it requires an aromatic ring in the 3-position as its absence causes it to shut down.

**Scheme 44:**
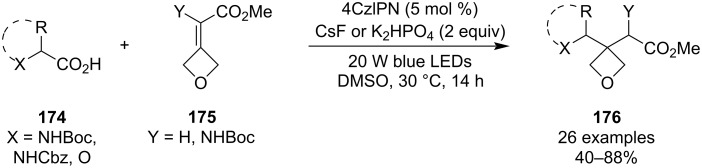
Synthesis of oxetane-amino esters **176**.

In 2024, Zhang and co-workers exploited the high reactivity of activated 3-alkylideneoxetanes **175** in a divergent synthesis of 3,3-disubstituted oxetane amino esters **176** based on a facile, strain-release-driven Giese addition of nitrogen- or oxygen-stabilised radicals (Scheme 44) [96]. The radicals were generated through a photochemical oxidative decarboxylation of amino- or alkoxycarboxylic acids under blue-light irradiation, and the Giese adducts were obtained in moderate to high yields. As demonstrated by the authors, this protocol is also suitable for synthesising oxetane-containing dipeptides, and opens the door for exploring a novel oxetane-spirocycle motif if the γ-aminoester product (**176**, X = NHBoc or NHCbz) is allowed to cyclise into a lactam.

### 3 Reactions of oxetanes

#### 3.1 Ring-opening reactions

The inherent ring strain of oxetanes and its polarised C–O bonds allow for relatively facile ring-opening reactions, typically upon a Lewis-acid activation. This type of reactivity has been greatly exploited in the past [34] and new protocols are still being developed and reported. These reactions can be divided into intramolecular and intermolecular processes and they usually rely on oxygen- or nitrogen-based nucleophiles, but ring openings involving hydrides and soft carbon nucleophiles have also been reported. The following sub-chapters will describe the recently developed systems, including possible mechanisms and potential applications, if appropriate.

**Scheme 45:**
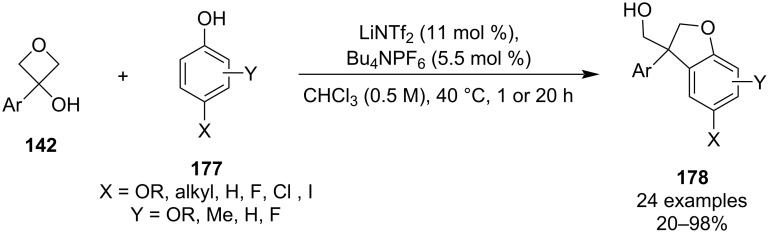
Tandem Friedel–Crafts alkylation/intramolecular ring opening of 3-aryloxetan-3-ols.

**3.1.1 Intramolecular ring-opening reactions:** In 2016, Bull and colleagues developed a mild approach towards 2,3-dihydrobenzofurans **178** from 3-aryloxetan-3-ols through a tandem Friedel–Crafts alkylation/intramolecular ring opening (Scheme 45) [87]. The reaction was mostly high yielding and best results were obtained for electron-rich *para*-substituted phenols, while substituents in the *ortho*/*meta*-positions diverted the regioselectivity of the Friedel–Crafts alkylation to the *para*-position, forming exclusively (or significant amounts of) 3,3-diaryloxetanes.

**Scheme 46:**
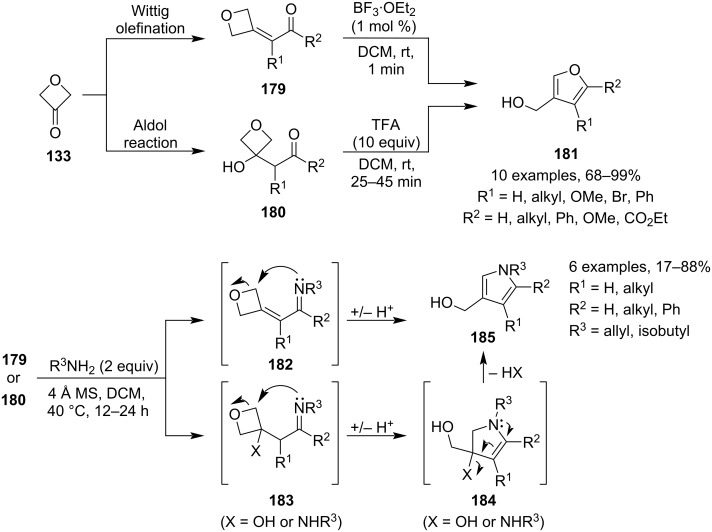
Synthesis of polysubstituted furans and pyrroles.

In 2017, Vanderwal and co-workers published a novel, facile synthesis of furans **181** and pyrroles **185** from 3-(2-oxoalkylidene)oxetanes **179**, precursors which are readily prepared by a Wittig olefination of commercially available 3-oxetanone (Scheme 46) [97]. As for the furan formation, the methodology employs ambient reaction conditions, super-short reaction times and very low catalyst loadings. During the reaction, the oxetane is activated by BF_3_, opened by the carbonyl oxygen and a subsequent proton transfer affords the aromatic heterocycle. The authors also devised a modification to this procedure for olefin precursors which were difficult to prepare: this alternative uses cross-aldol adducts **180** between 3-oxetanone and a ketone, and the ring opening and dehydration (necessary for aromatisation) are promoted by trifluoroacetic acid under similarly mild conditions. In case of the pyrrole synthesis, the same precursors are treated with a primary amine in DCM and the reaction mixture is heated at 40 °C for 12–24 h. As 1,4-addition also takes place during this process, two equivalents of the amine and longer reaction times were found necessary to transform the undesired adducts **183** (X = NHR^3^) to the pyrroles and hence obtain higher yields.

**Scheme 47:**
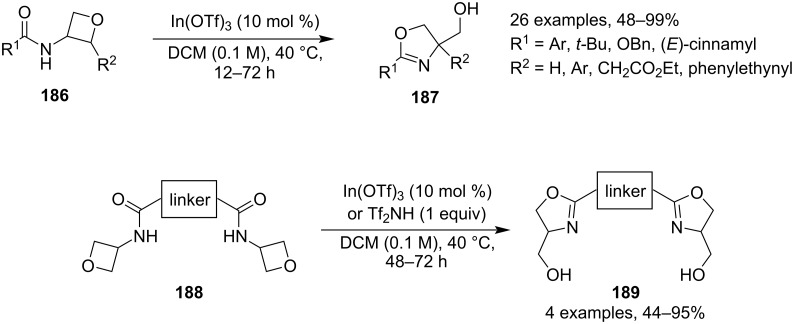
Synthesis of oxazolines and bisoxazolines.

Two years later, Sun and co-workers reported a convenient and highly efficient synthesis of 2-oxazolines **187** which applied mild conditions, readily available oxetanes and only catalytic amounts of the activator (Scheme 47) [98]. The reaction is based on activating *N*-acyl-3-aminooxetanes **186** with indium triflate in refluxing dichloromethane to trigger the opening of the oxetane by the acyl oxygen. The methodology gave excellent yields in most cases and worked very well for both aromatic and aliphatic amides, as well as sterically hindered oxetanes. The authors further proved the robustness of this reaction by preparing various bisoxazolines **189**, compounds which are common bidentate ligands in asymmetric catalysis [99].

**Scheme 48:**
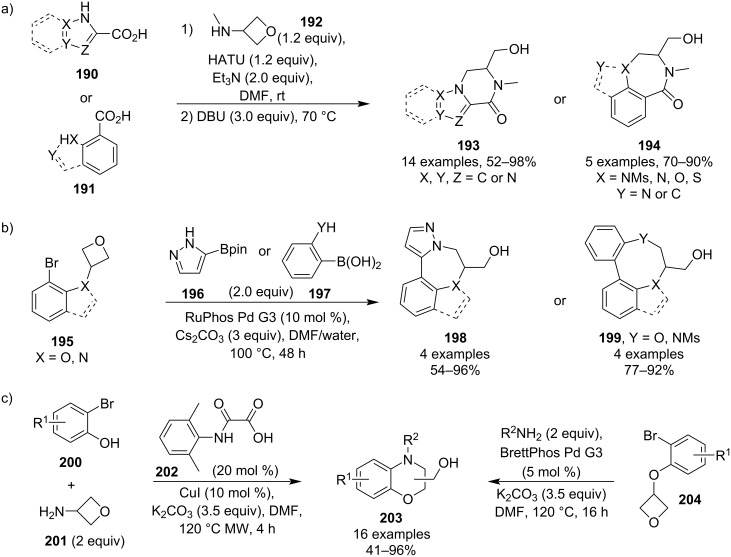
Tandem, one-pot syntheses of various polycyclic heterocycles.

Over the years 2019–2021, Kuduk and co-workers published three different tandem, one-pot methodologies towards complex polycyclic heterocycles [100–102]. All of them are two-step reactions consisting of an intermolecular coupling followed by an intramolecular, base-induced oxetane opening (Scheme 48). The first one utilised a HATU-promoted amide formation involving 3-(methylamino)oxetane (**192**) in the first step and the resulting bi- and tricyclic products **193** and **194** containing 6- and 7-membered rings were obtained in high yields (Scheme 48a). The second methodology generated similar polycyclic systems but used a Suzuki coupling to tether the oxetane with the nucleophile, and gave moderate to high yields (Scheme 48b). The last one employed an Ullman or Buchwald–Hartwig coupling and provided access to various benzomorpholines **203** with good functional group tolerance (Scheme 48c).

**Scheme 49:**
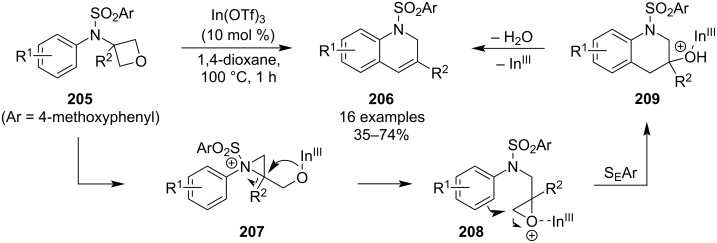
Synthesis of 1,2-dihydroquinolines via skeletal reorganisation of oxetanes.

In 2020, Sun et al. reported a novel synthesis of 1,2-dihydroquinolines **206** by an unprecedented skeletal reorganisation of oxetanes (Scheme 49) [103]. The reaction employs oxetane-tethered electron-rich sulphonamides **205** and is triggered by indium triflate at an elevated temperature. The products were obtained in moderate yields and could be further aromatized to quinolines upon treatment with ethanolic sodium hydroxide at 100 °C. Subsequent mechanistic studies showed an initial oxetane opening by the sulphonamide nitrogen as the most probable pathway.

**Scheme 50:**
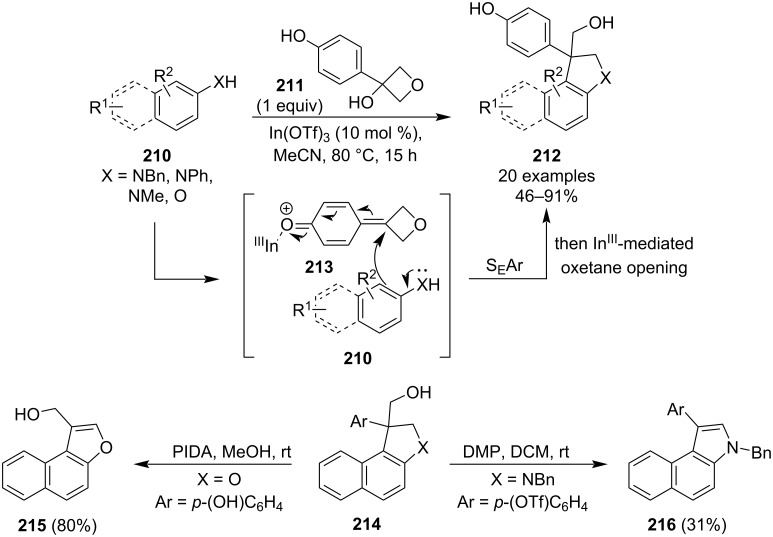
Synthesis of benzoindolines and 2,3-dihydrobenzofurans and their derivatisations.

Seven months later, the same research group published another indium triflate-catalysed heterocycle synthesis, specifically a [3 + 2] annulation of 3-(4-hydroxyphenyl)oxetan-3-ol (**211**) with β-naphthylamines or phenols **210** to give benzoindolines and 2,3-dihydrobenzofurans **212**, respectively (Scheme 50) [104]. The reaction gave moderate to high yields and control experiments indicated that it likely proceeds through a *p*-quinone methide intermediate **213**. The authors also demonstrated on two selected examples (**214**) that the products could be further derivatised to 3-substituted benzofurans **215** and benzindoles **216** with a hypervalent iodine reagent.

**Scheme 51:**
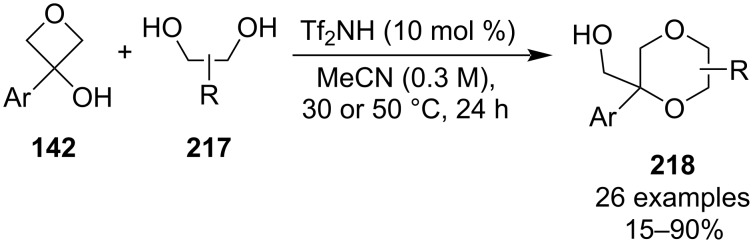
Synthesis of polysubstituted 1,4-dioxanes.

In 2022, Bull et al. disclosed a metal-free annulation reaction between electron-rich 3-aryloxetan-3-ols **142** and 1,2-diols under Brønsted acid catalysis (Scheme 51) [105]. The resulting 1,4-dioxanes **218** were obtained mostly in moderate to high yields and high regio- and diastereoselectivities were achieved with unsymmetrical diols. The reaction is assumed to proceed via an initial S_N_1 substitution of the tertiary alcohol followed by intramolecular opening of the oxetane ring. The authors further demonstrated the wide scope of this methodology by preparing spirocyclic and fused bicyclic dioxanes as well as sulphur-containing heterocycles.

**Scheme 52:**
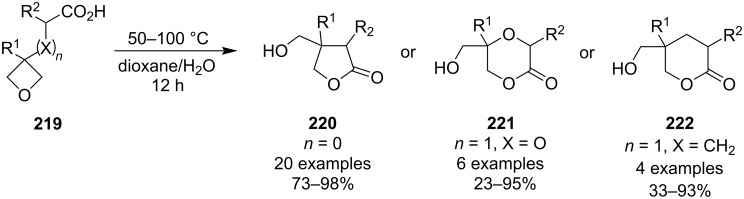
Preparation of various lactones via ring opening of oxetane-carboxylic acids **219**.

In the same year, Mykhailiuk and co-workers exploited the inherent propensity of oxetane-carboxylic acids **219** for intramolecular ring openings to generate functionalised γ-butyrolactones **220**, dioxanones **221** and valerolactones **222** (Scheme 52) [106]. The conditions of the reported method involve only simple heating in aqueous dioxane and the lactone products were obtained in moderate to excellent yields.

**Scheme 53:**
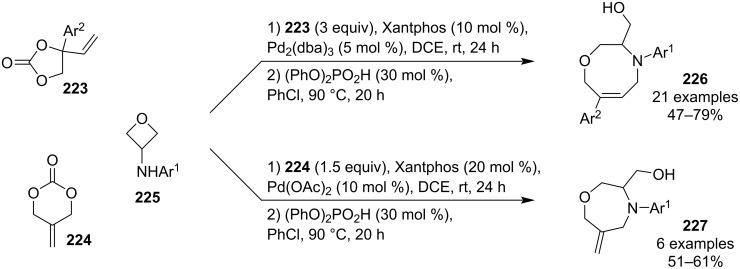
Tsuji-Trost allylation/ring opening of 3-aminooxetanes.

In 2023, Han and Huang et al. developed a two-step protocol for the synthesis of tetrahydro-2*H*-1,4-oxazocines **226** and 1,4-oxazepanes **227** by a Tsuji–Trost allylation of 3-(arylamino)oxetanes **225** followed by an acid-catalysed intramolecular oxetane opening (Scheme 53) [107]. Although it is not a true domino process as the ring opening does not take place in situ, the work-up after the first step involves only a short column filtration and concentration, and the target medium-size heterocycles are obtained in good overall yields.

**Scheme 54:**
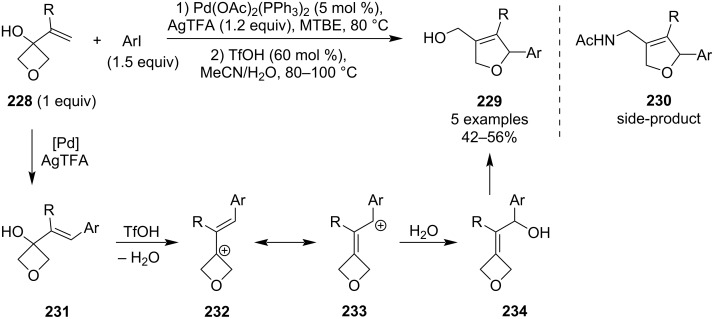
Arylative skeletal rearrangement of 3-vinyloxetan-3-ols to 2,5-dihydrofurans.

In 2024, Zhu et al. published a dual Pd and acid-catalysed arylative skeletal rearrangement of 3-vinyloxetan-3-ols **228** to 2,5-dihydrofurans **229** (Scheme 54) [108]. Although this transformation was originally developed for azetidine analogues, which required a 1-step procedure and smaller catalyst loadings, further tuning of the reaction conditions (to those shown in Scheme 54) allowed for a facile transformation of oxetanes as well, with five examples being reported. However, in case of these oxetane substrates, it is important not to extend the reaction time too much as the allylic alcohol in the dihydrofuran product will get substituted by acetonitrile and subsequent hydrolysis of the nitrilium ion will deliver acetamide **230**. The mechanistic proposal involves a Heck arylation, acid-catalysed transposition of the allylic hydroxy group and ring opening of the oxetane.

**Scheme 55:**
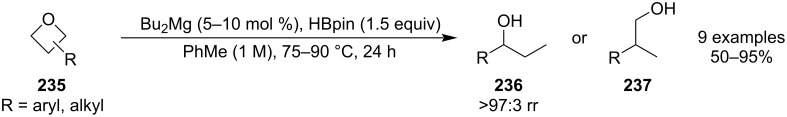
Reductive opening of oxetanes using catalytic Mg–H species.

**3.1.2 Intermolecular ring-opening reactions:** Significant progress has also been achieved in intermolecular ring-opening reactions of oxetanes, particularly in developing very mild reaction conditions for reductive opening and C–C-bond forming reactions, as well as unprecedented transition-metal-catalysed transformations. In 2020, Rueping and colleagues published a catalytic and highly regioselective method for the reductive opening of monosubstituted oxetanes using a combination of dibutylmagnesium and pinacolborane (Scheme 55) [109]. These reagents form a butylmagnesium hydride species in situ which then regioselectively reduces the sterically more accessible C–O bond of the oxetane (in case of 2-substituted oxetanes). Subsequent metathesis of the Mg–O and B–H bonds regenerates the active reducing agent and the free alcohol product **236** or **237** is liberated from the borate by a methanol quench.

**Scheme 56:**
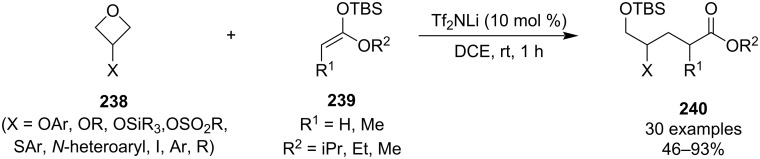
Opening of oxetanes by silyl ketene acetals.

In the same year, Sun and co-workers reported a very mild synthesis of δ-hydroxyesters **240** based on a metal-catalysed oxetane opening, which constitutes a versatile alternative to the Michael addition of enolates (Scheme 56) [110]. In addition, the protocol used soft carbon nucleophiles **239** and a relatively weak activator that exhibited excellent chemoselectivity. This represents a major breakthrough as previous methods often relied on highly reactive nucleophiles and activators, such as RLi and BF_3_, that have poor functional group compatibility.

**Scheme 57:**
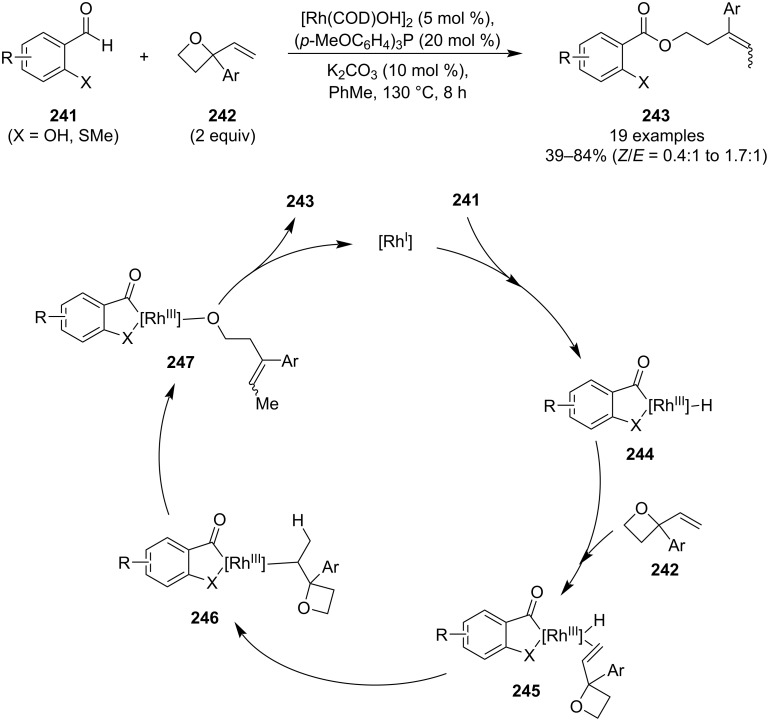
Rhodium-catalysed hydroacylation of oxetanes.

In 2021, Li et al. disclosed an unprecedented rhodium-catalysed hydroacylation of 2-aryl-2-vinyloxetanes **242** using chelating benzaldehydes **241** (Scheme 57) [111]. The resulting esters **243** were obtained in mostly moderate yields, bearing an “alkene handle” for further functionalisation, and in a completely atom-economical manner. However, this protocol suffers from low selectivity in terms of the alkene geometry. Mechanistic studies suggested the following sequence of steps: oxidative addition of Rh(I) to the aldehyde, alkene coordination and insertion, oxetane opening and reductive elimination.

**Scheme 58:**
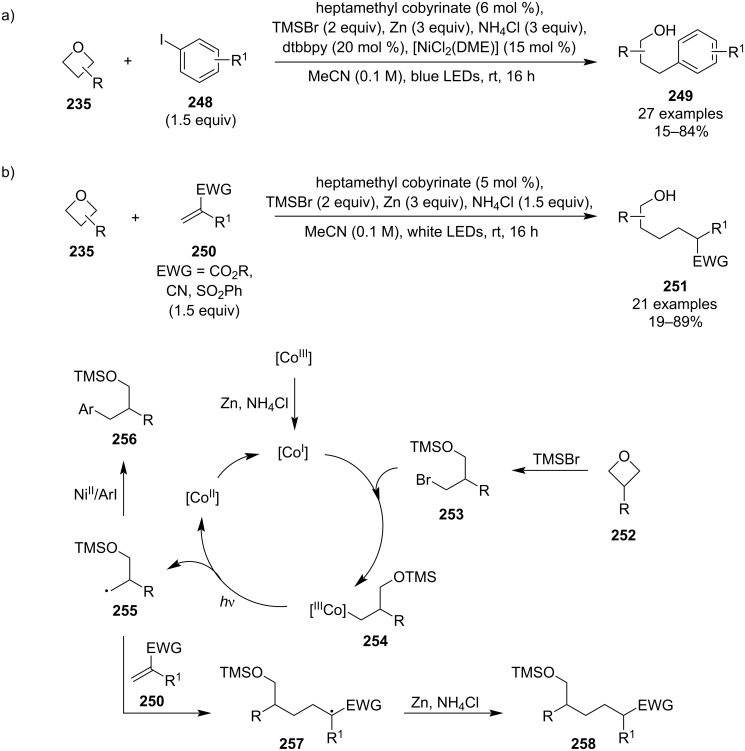
Generation of radicals from oxetanes mediated by a vitamin B_12_-derived cobalt catalyst.

In 2022, another unprecedented transition-metal-mediated opening of oxetanes emerged using a vitamin B_12_-derived cobalt catalyst. This methodology, developed by Gryko et al., constitutes the first example of generating synthetically useful radicals from oxetanes (Scheme 58) [112]. The generated radical intermediates **255** were reacted in two different modes and the research group optimised the reaction conditions for each one: a) the radicals entered a cross-electrophile coupling catalytic cycle mediated by nickel, or b) the radicals were trapped by electron-deficient alkenes in a Giese-type reaction. Both modes gave mainly moderate yields and tolerated various functional groups such as (hetero)aryls, alkyls or protected alcohols and amines. A general catalytic cycle was proposed for the radical generation which involved reduction of the cobalt catalyst by zinc, oxetane opening by TMSBr to give a silyl-protected bromoalcohol **253**, oxidative addition to the Co(I) complex and light-induced homolytic cleavage of the Co–C bond. The final alcohol desilylation was achieved by an acidic work-up.

**Scheme 59:**
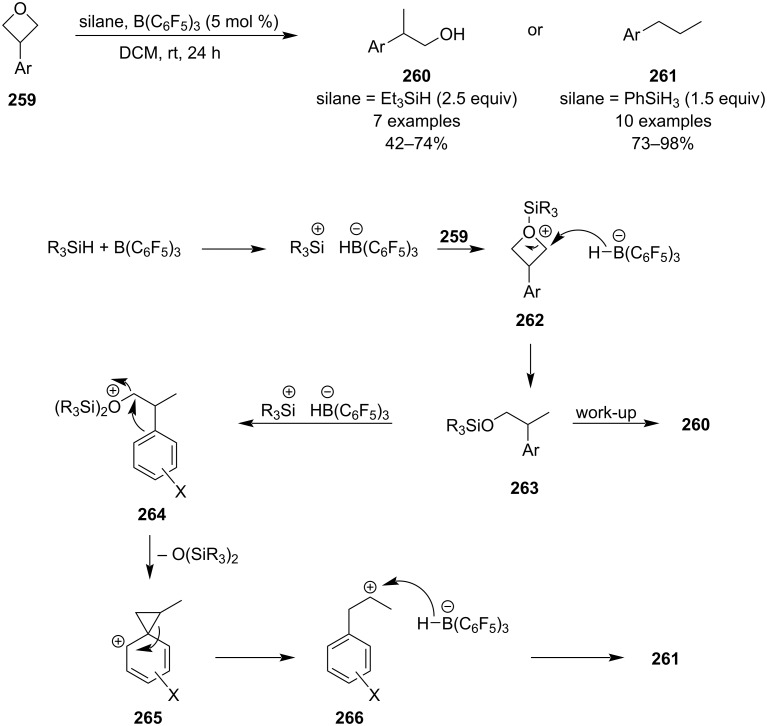
Reductive opening of oxetanes by B–Si frustrated Lewis pairs.

In the same year, Sun and colleagues reported a super-mild protocol for the reductive opening of 3-aryloxetanes **259** utilising frustrated Lewis pair chemistry (Scheme 59) [113]. Depending on the reductant used, the reaction proceeds either through classical reduction affording 2-arylpropan-1-ols **260** or through aryl migration/deoxygenation to give 1-arylpropanes **261**. The protocol gave low to moderate yields for the former pathway but high to excellent yields for the latter, and proceeded well in the presence of various functional groups such as alkynes, alkenes or heterocycles. Control experiments suggested an involvement of phenonium intermediate **265** in the aryl migration pathway and thus a plausible mechanism was proposed.

**Scheme 60:**
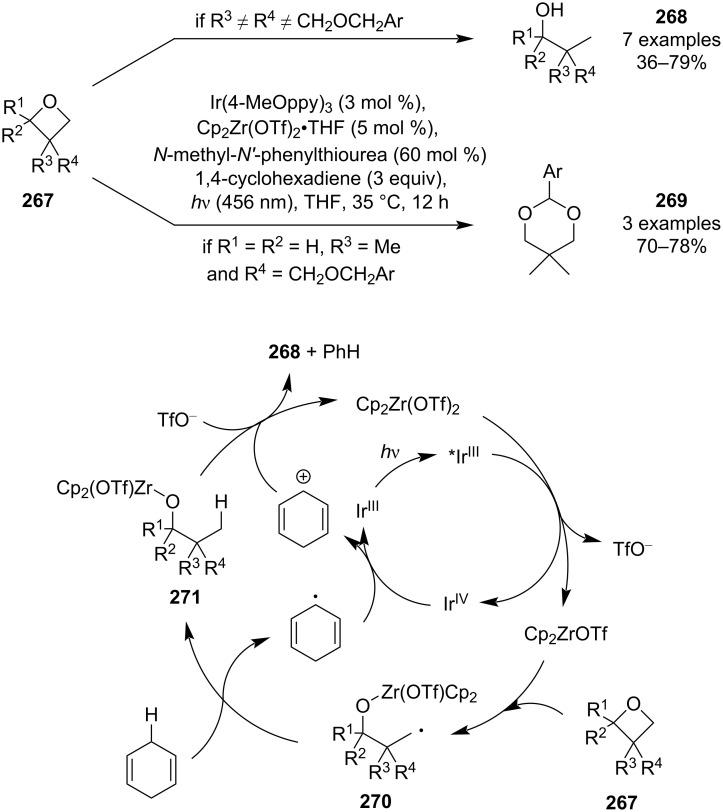
Zirconocene-mediated reductive opening of oxetanes.

In 2023, Ota, Yamaguchi and colleague disclosed a novel, highly regioselective reductive opening of oxetanes based on a homolytic cleavage of the C–O bond at the less-substituted carbon (Scheme 60) [114]. The protocol combines photoredox and zirconocene catalysis in which the zirconium metal plays a crucial role in achieving this unique regioselectivity as it forms a strong Zr–O bond, thus leading to an early transition state for the ring opening [115]. Various functional groups were tolerated, such as ketones, esters, amides, ethers or chloroalkanes, but in case of 3-(benzyloxymethyl)oxetanes, formation of cyclic acetals **269** through 1,5-HAT and cyclisation was observed. Mechanistic studies were not conducted but the authors implied that the mechanism is analogous to the one proposed in their previous work on reductive opening of epoxides [115].

**Scheme 61:**
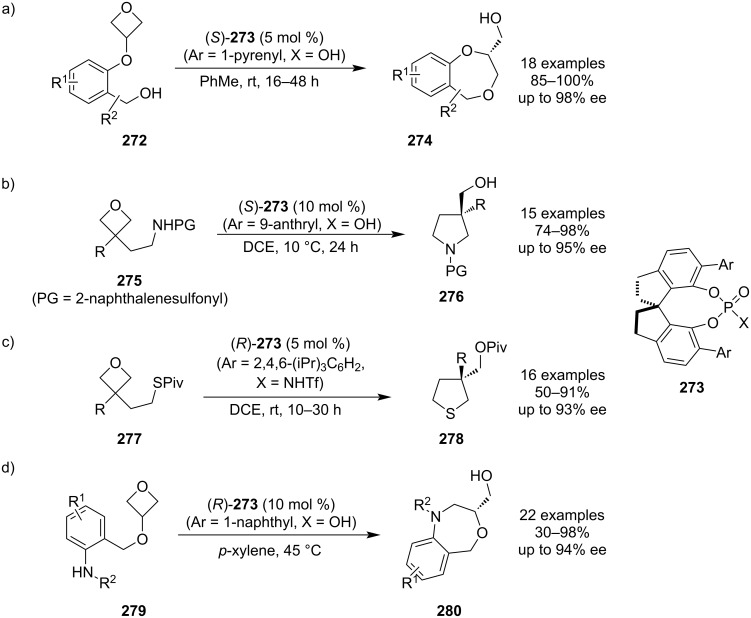
Enantioselective syntheses of small and medium-size rings using chiral phosphoric acids.

**3.1.3 Enantioselective ring openings of 3-substituted oxetanes:** The first example of an asymmetric synthesis based on oxetane desymmetrisation was reported by Tomioka et al. in 1997 who treated 3-phenyloxetane with organolithium reagents in the presence of chiral ethers [116]. Since then, new strategies have been emerging with different types of the chiral activator including Brønsted acids, Lewis acids, sulphinamides or hydrogen-bond-donor catalysts such as squaramides. In the past few years, great progress has been achieved by Sun and colleagues who developed protocols for catalytic asymmetric syntheses of small and medium-size rings using chiral phosphoric acids possessing the common chiral backbone **273** (Scheme 61a–c): a) The phosphoric acid (X = OH) bearing 1-pyrenyl as the aryl group was utilised for the preparation of 1,4-benzodioxepines **274** under mild conditions in almost quantitative yields and with excellent enantioselectivities (ee up to 98%) [117]; b) The acid analogue (X = OH) bearing 9-anthryl was found to be a suitable catalyst for a convenient synthesis of chiral 3,3-disubstituted pyrrolidines **276**, whose synthesis had been rather challenging, in very high yields and with ees about 90% [118]; c) The catalyst possessing the triflimide moiety (X = NHTf) and 2,4,6-triisopropylphenyl as the aryl group was found to effectively catalyse an asymmetric synthesis of tetrahydrothiophenes **278** under ambient conditions, but in somewhat lower yields and ees [119]. The optimisation studies revealed the success of this latter reaction depended on protecting the thiol nucleophile as thioester **277** and mechanistic studies suggested that the acyl group is transferred intramolecularly. In 2023, Veselý et al. further expanded the substrate scope of this methodology and developed a highly enantioselective synthesis of 1,4-benzoxazepines using the phosphoric acid catalyst (X = OH) containing 1-naphthyl substituents (Scheme 61d) [120].

**Scheme 62:**
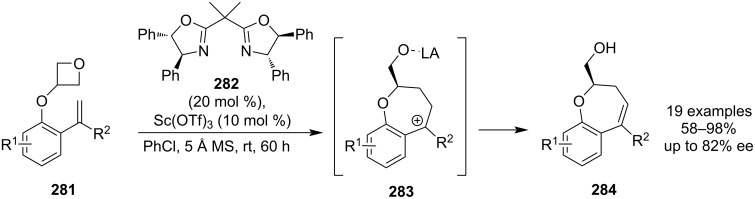
Asymmetric synthesis of 2,3-dihydrobenzo[*b*]oxepines catalysed by a chiral scandium complex.

Catalytic asymmetric oxetane openings by carbon-based nucleophiles are also known and a new example emerged in 2022: this unprecedented methodology uses a chiral scandium-based Lewis acid to promote the generation of 2,3-dihydrobenzo[*b*]oxepines **284** from 3-(2-vinylaryloxy)oxetanes **281** (Scheme 62) [121]. However, anhydrous conditions and prolonged reaction times are necessary to obtain high yields and the enantioselectivities were only moderate (ee up to 82%). The reaction is believed to proceed via a benzylic carbocation **283**.

**Scheme 63:**
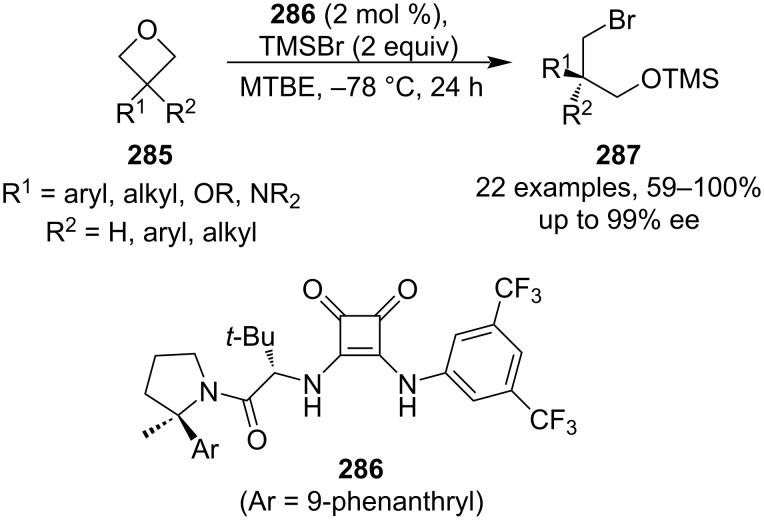
Enantioselective synthesis of 1,3-bromohydrins under a chiral squaramide catalysis.

As for asymmetric intermolecular oxetane openings, a new protocol was reported in 2020 by the Jacobsen group. This protocol uses chiral squaramide catalyst **286** in the TMSBr-promoted oxetane opening to afford enantioenriched 1,3-bromohydrins **287** (Scheme 63) [122]. Although strong cooling over 24 h is necessary, the reaction is very high yielding, provides excellent enantioselectivities and tolerates various functional groups including aryls, alkyls or protected alcohols and amines. In addition, this method constitutes a respectable alternative to glycidol- or epichlorohydrin-based syntheses of chiral 3-carbon building blocks.

**Scheme 64:**
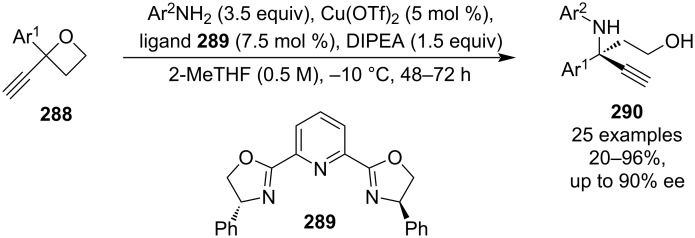
Enantioselective opening of 2-aryl-2-ethynyloxetanes by anilines.

In 2024, Kleij et al. reported the first asymmetric synthesis of γ-amino alcohols containing a tertiary carbon stereocentre based on a Lewis-acid-catalysed opening of 2-aryl-2-ethynyloxetanes **288** by anilines (Scheme 64) [123]. The oxetanes are accessible in 5 simple steps from the corresponding benzaldehydes requiring just one purification, and enantioselectivity is controlled by the copper complex formed by reacting Cu(OTf)_2_ and bisoxazoline **289**. The ring openings proceeded mostly in good to high yields and enantiomeric ratios, various substituents and substitution patterns were tolerated, and some of the products were further modified for example by a Sonogashira coupling, esterification or intramolecular alkynylation to demonstrate their synthetic potential.

#### 3.2 Ring-expansion reactions

**Scheme 65:**
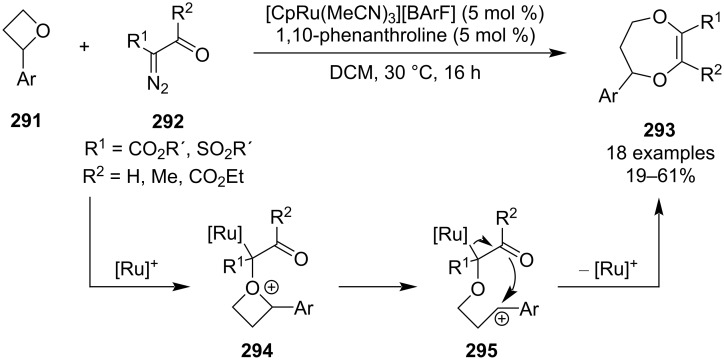
Ru-catalysed insertion of diazocarbonyls into oxetanes.

Besides ring openings, the inherent ring strain in oxetanes also allows for relatively facile carbon-based fragment insertions and rearrangements, ultimately leading to an expansion of the ring and relieving the strain. These reactions can be induced by light, transition metals or strong acids. In 2017, Lacour and co-workers developed a synthesis of 1,4-dioxepines **293** based on a novel Ru-catalysed [4 + 1] insertion of 2-diazo-1,3-dicarbonyls into 2-aryloxetanes (Scheme 65) [124]. The reaction conditions are very mild and the dioxepines were obtained as single regioisomers in low to moderate yields. According to the proposed mechanism, the metallacarbene formed upon nitrogen elimination reacts with the oxetane to form a metal-bound oxonium ylide **294**, which, due to the ring strain and stabilising effect of the aryl, undergoes heterolytic C–O-bond cleavage to afford carbocation **295**. Subsequent rapid 7-*endo*-*trig* cyclisation through the carbonyl oxygen accompanied by ruthenium dissociation delivers the 7-membered heterocycle. Although the mechanism is largely S_N_1-like, the fast and intramolecular trapping of the carbocation ensures partial retention of the configuration (es 74%) present in the oxetane, which was supported by vibrational circular dichroism and X-ray diffraction analyses.

**Scheme 66:**
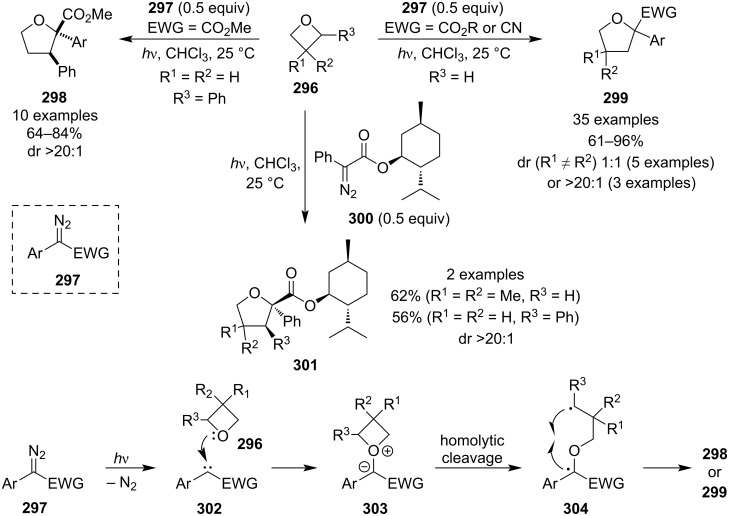
Ring expansion of oxetanes by stabilised carbenes generated under blue light irradiation.

In 2019, Koenigs et al. published a ring expansion of oxetanes using photochemically generated stabilised carbenes to afford tetrahydrofurans **298** and **299** (Scheme 66) [125]. This protocol is particularly convenient as it does not require any additives, proceeds at room temperature, affords high yields and diastereoselectivities (for chiral oxetanes substituted at position 2) and does not lead to overreaction. Moreover, the authors found that the diastereoselectivity could also be controlled by using menthol esters such as **300**. DFT calculations suggested that the reaction proceeds via nitrogen elimination, oxonium ylide **303** formation, homolytic cleavage and radical recombination.

**Scheme 67:**
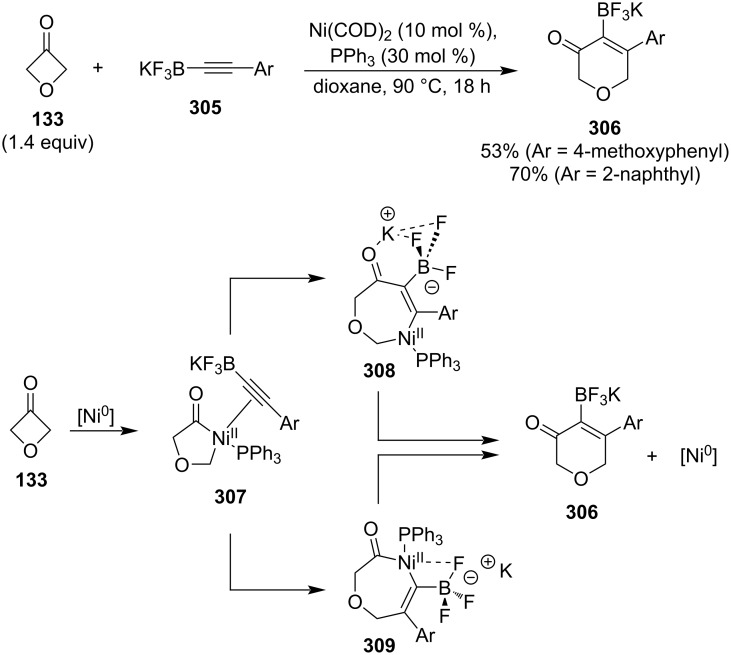
Expansion of oxetanes via nickel-catalysed insertion of alkynyltrifluoroborates.

Aïssa and co-workers developed a novel nickel-catalysed insertion of alkynyltrifluoroborates **305** into 4-membered heterocyclic ketones, including 3-oxetanone (Scheme 67) [126]. The key features of this reaction are complete regioselectivity, which is not affected by the alkyne substituent, and retention of the trifluoroborate group which can serve as a handle for subsequent cross-coupling reactions. The authors presumed that the two most contributing mechanistic pathways involve oxidative addition of nickel into the oxetanone, alkyne coordination, migratory insertion controlled by interactions of either the potassium cation with the carbonyl oxygen (**308**) or a fluorine atom with the nickel (**309**), and reductive elimination.

**Scheme 68:**
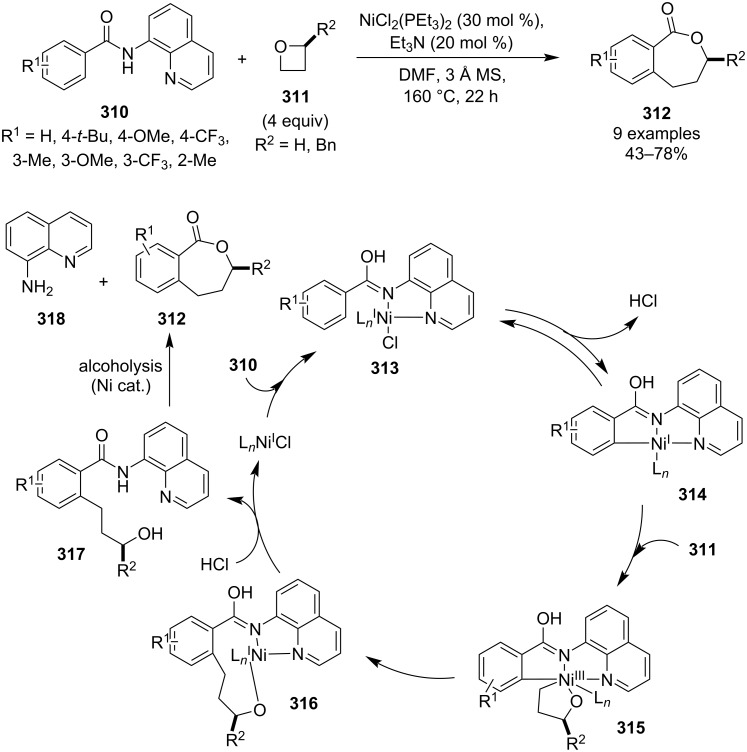
Nickel-catalysed expansion of oxetanes into ε-caprolactones.

Miura and colleagues disclosed in 2019 a nickel-catalysed C–H coupling of benzamides **310** with oxetanes to generate 7-membered benzolactones **312**, which represent a common structural motif in natural products and bioactive molecules (Scheme 68) [127]. Regioselectivity of the C–H activation is governed by a chelating 8-aminoquinoline auxiliary, which is cleaved off at the end of the catalytic cycle. The products were obtained in moderate yields and besides monosubstituted phenyls (bearing the amide group), the scope was also extended to naphthalene, thiophene, pyrrole and indole. Furthermore, retention of chirality was observed when employing an enantioenriched 2-substituted oxetane as the reaction proceeds at the sterically more accessible side of the substrate. Mechanistic studies indicated that the reaction is actually catalysed by a Ni(I) species probably formed by a reduction with the phosphine. Subsequent chelation, C–H activation and oxidative addition afford complex **315**, which upon reductive elimination and protonolysis forms alcohol **317** and regenerates the catalyst. Finally, intramolecular alcoholysis, possibly catalysed by nickel, generates the benzolactone product.

**Scheme 69:**
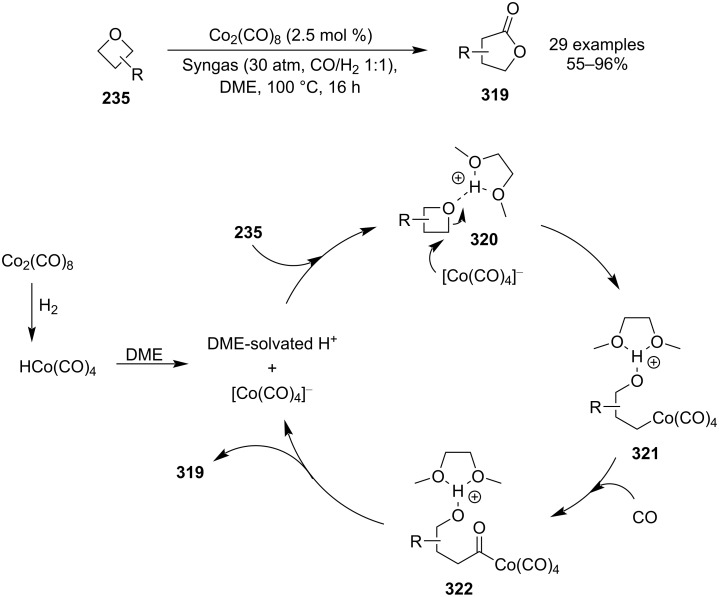
Expansion of oxetanes via cobalt-catalysed carbonyl insertion.

In 2020, Dong et al. reported an efficient cobalt-catalysed synthesis of γ-lactones **319** via carbonyl insertion into substituted oxetanes **235** under a Syngas atmosphere (Scheme 69) [128]. Although the practicality of this protocol in a laboratory might not be ideal due to the high pressures of toxic CO and explosive hydrogen gas, it might be well-suited for industry, particularly due to the relatively low costs of the chemicals, straightforward route and high atom economy. The authors also demonstrated these advantages by applying their protocol in a multi-gram synthesis of a precursor for the asthma treatment medicine montelukast. Computational studies support the following mechanism: hydrogenation of the pre-catalyst followed by dissociation into the anionic [Co(CO)_4_]^−^ complex and DME-solvated proton, H^+^-catalysed oxetane opening by the negatively charged cobalt (**320**), CO insertion (**322**) and lactonisation.

**Scheme 70:**
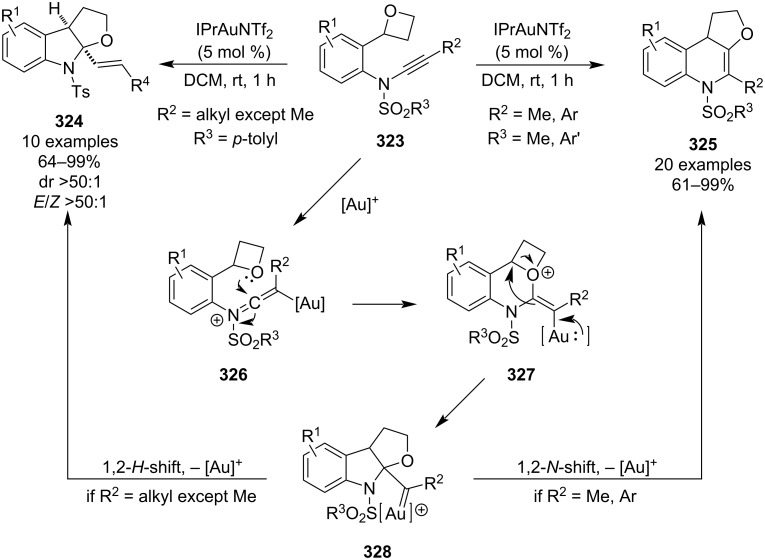
Gold-catalysed intramolecular 1,1-carboalkoxylation of oxetane-ynamides.

In 2021, Ye and co-workers published an unprecedented gold-catalysed intramolecular 1,1-carboalkoxylation of oxetane-ynamides **323** (Scheme 70) [129]. Depending on the substituent attached to the alkyne, either tetrahydrofuran-fused 1,4-dihydroquinolines **325** or furoindolines **324** were generated. In both cases, the reaction proceeds at room temperature, with short reaction times and in very high yields. In case of the furoindolines **324**, it also exhibits excellent diastereo- and *E*/*Z*-selectivities and allows for a complete chirality transfer if enantioenriched oxetanes are used. Alternatively, chiral phosphoramide ligands can be used to kinetically resolve racemic substrates to achieve enantioselectivities up to 56% ee. Based on the stereochemical outcomes and ensuing DFT calculations, the authors proposed the following mechanism: gold-mediated 6-*endo*-*dig* cyclisation (**326**), oxetane opening (**327**) and 1,2-*H*-shift (for alkyl substituents except for methyl) or 1,2-*N*-shift (for methyl or aryl substituents).

**Scheme 71:**
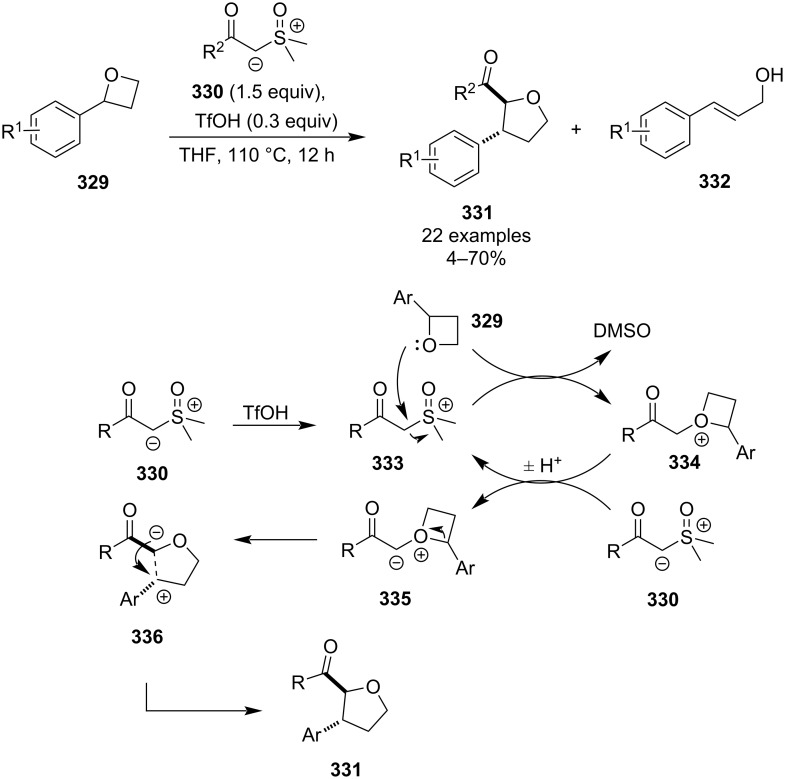
Expansion of oxetanes by stabilised sulphoxonium ylides.

In the same year, Xu and colleagues disclosed a strong-acid-promoted ring expansion of 2-aryloxetanes **329** with stable sulphoxonium ylides **330** to afford *trans*-2,3-disubstituted tetrahydrofurans **331** (Scheme 71) [130]. Although the reaction proceeds stereospecifically without a metal catalyst and protective atmosphere, it suffers from harsh reaction conditions and rather low yields due to the formation of cinnamyl alcohols **332** as side products. The yields get even lower for electron-poor aryl substituents which reduce the nucleophilicity of the oxetane and favour the side product formation via acid-catalysed elimination. The authors proposed a mechanism which starts with the protonation of the ylide and subsequent S_N_2 substitution of the DMSO component by the oxetane. The resulting oxonium species **334** then gets deprotonated by the sulphoxonium ylide **330** (whose conjugate acid **333** re-enters the cycle) to give oxonium ylide **335** which undergoes ring opening to generate zwitterion **336**. Finally, a C–C-bond forming step via the thermodynamically favoured *trans*-disubstituted 5-membered transition state affords the tetrahydrofuran product **331**.

**Scheme 72:**
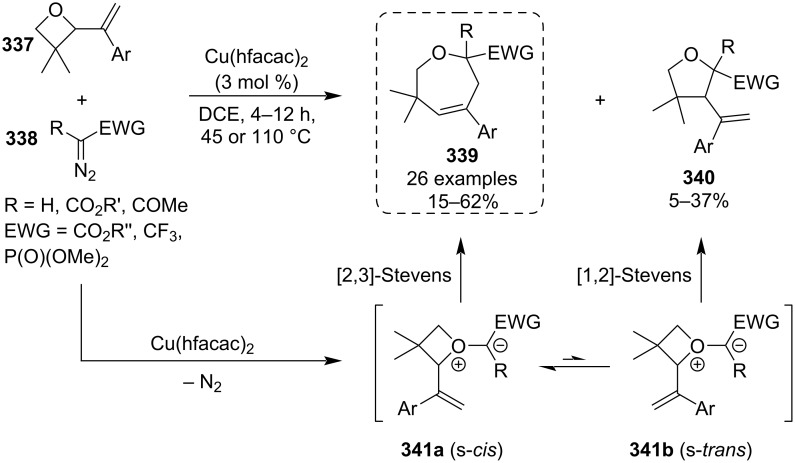
Cu-catalysed ring expansion of 2-vinyloxetanes by diazoesters.

In 2024, Lacour and co-workers published an efficient synthesis of tetrahydrooxepines **339** by reacting oxetanes **337** with copper-stabilised carbenes generated in situ from diazo reagents **338** (Scheme 72) [131]. The target 7-membered heterocycles were obtained in low to moderate yields due to a competing side reaction producing tetrahydrofurans **340**, which were in the vast majority of cases the minor products mainly due to the *gem*-dimethyl effect exerted by the oxetane. Also, employing the more reactive monofunctionalised diazo compounds (R = H) further suppressed formation of **340** to trace amounts and allowed the reactions to proceed at 45 °C. The reaction mechanism is believed to consist of oxonium ylide **341a**,**b** formation and either [2,3]- or [1,2]-Stevens rearrangement depending on the ylide conformation, where the s-*cis* conformation is preferred due to the *gem*-dimethyl functionality.

### 4 Total syntheses of oxetane-containing natural products

#### 4.1 Oxetin

**Scheme 73:**
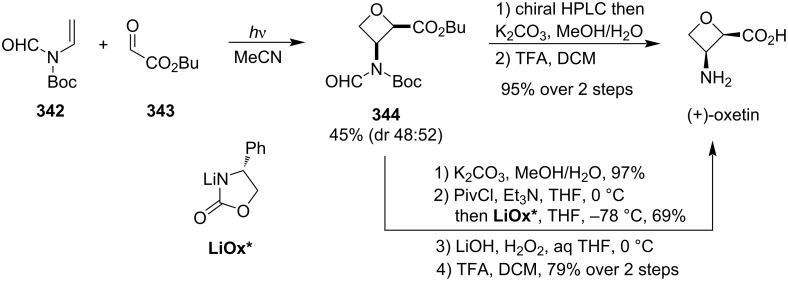
Total synthesis of (+)-oxetin.

Oxetin (Scheme 73) is a cyclic β-amino acid isolated in 1984 from the soil bacterium *Streptomyces sp*. OM-2317 possessing antibiotic and herbicidal activity [23]. It bears the (2*R*,3*S*) configuration, corresponding to the *cis* diastereomer, and it is the first reported natural product containing an oxetane ring. Despite its relatively simple structure, the first total synthesis via Williamson etherification and oxidative diol cleavage from glucose required more than 10 steps [132]. However, the synthetic sequence got significantly shortened upon utilisation of the Paternò–Büchi reaction and the most recent total synthesis by Aitken et al. consists of only 3 or 5 steps depending on the enantiomeric resolution (Scheme 73) [133]. The key photocycloaddition was conducted between Boc-protected *N*-vinylformamide **342** and butyl glyoxylate (**343**) on a multigram scale affording the disubstituted oxetane **344** as an almost 1:1 mixture of separable diastereomers in 45% yield. Then, two different methods were developed for the resolution of the enantiomers: a) the formamide and ester were hydrolysed and the free carboxylic acid was coupled with chiral oxazolidinone LiOx* – subsequent chromatographic separation, removal of the chiral auxiliary and Boc deprotection afforded the target natural product; b) the enantiomeric oxetanes **344** were separated by chiral HPLC on a lux cellulose column and subsequent global deprotection gave oxetin. The key highlights of this synthesis are scalability, producing oxetin on a gram-scale, small number of steps and versatility, providing access to all four stereoisomers of the natural product in a roughly 10% overall yield for a single isomer.

#### 4.2 Oxetanocin A

**Scheme 74:**
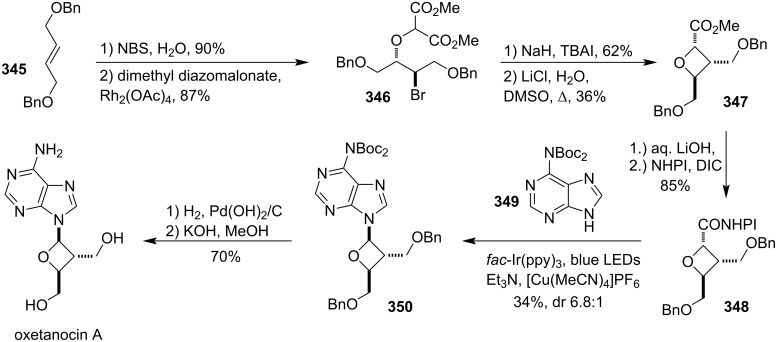
Total synthesis of racemic oxetanocin A.

Isolated in 1986 from the bacterial strain *Bacillus megaterium* NK84-0218, this nucleoside analogue possesses interesting antibiotic and antiviral activities, for example against *Staphylococcus aureus* 209P, herpes simplex virus-II or HIV-1 [22,134]. Besides the promising bioactivity, it is also the first natural nucleoside possessing an oxetane which attracted the attention of many synthetic chemists, and by 1990 five total syntheses were reported [135]. The most recent one was disclosed by Yang, Li and colleagues in 2024 which utilised a particularly mild *N*-glycosylation method based on a photoredox/Cu-catalysed decarboxylative coupling, specifically designed for acid-sensitive substrates such as the oxetanose moiety (Scheme 74) [136]. Starting from readily obtainable benzyl-protected diol **345**, bromohydroxylation followed by a Rh-mediated O–H insertion of the metallacarbene derived from dimethyl diazomalonate afforded bromodiester **346** in a high yield. Subsequent intramolecular substitution promoted by sodium hydride and Krapcho demethoxycarbonylation generated oxetane-monoester **347**, which was hydrolysed and the carboxylic acid condensed with *N*-hydroxyphthalimide (NHPI) in the presence of diisopropylcarbodiimide. The resulting NHPI ester **348** was then subjected to the developed *N*-glycosylation with Boc-protected adenine **349**: classical homolytic cleavage of the N–O bond of the NHPI group (caused by a single-electron transfer from the excited iridium photocatalyst) followed by decarboxylation generated a 2-oxetanyl radical which was coupled with the heterocycle in a Cu(I)/Cu(III) catalytic cycle, affording a 6.8:1 mixture of diastereomers in favour of the desired anomer **350**. After separation, the nucleoside was deprotected under acid-free conditions to provide racemic oxetanocin A in 70% yield.

#### 4.3 Merrilactone A

**Scheme 75:**
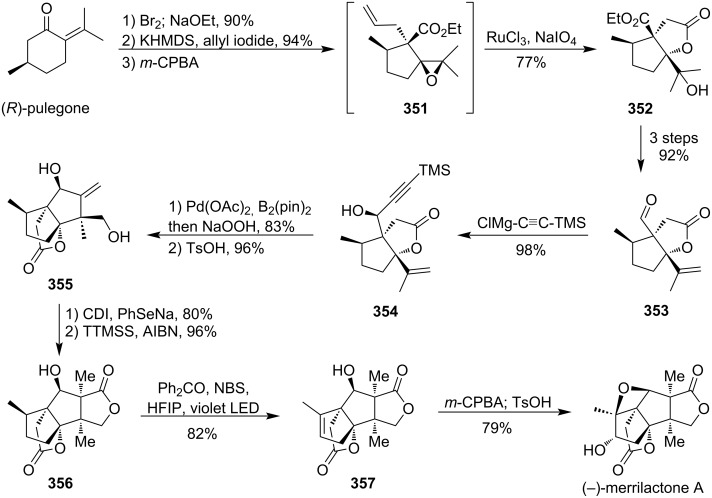
Total synthesis of (−)-merrilactone A.

This sesquiterpene (Scheme 75) was isolated from the pericarps of *Illicium merrillianum* in 2000 and displays a potent neurotrophic activity, greatly promoting neurite outgrowth [24]. Its intriguing biological activity as well as the challenging polycyclic structure prompted many chemists to develop a total synthesis of this natural product and these endeavours have culminated in 13 reported syntheses (total and formal). One of the most elegant was disclosed by Zhang and Zhang et al. in 2021 (Scheme 75) [137]. Their synthesis commenced with a Favorskii ring contraction of (*R*)-pulegone followed by allylation and regioselective epoxidation of the more substituted alkene (formed in the first step by bromide elimination) to afford epoxide **351**. Oxidative alkene cleavage with RuCl_3_/NaIO_4_ furnished a transient carboxylic acid which spontaneously opened the epoxide forming lactone **352**, and then a simple series of functional group interconversions and a stereoselective alkyne addition afforded enyne **354**. Palladium-catalysed borylative cycloisomerisation followed by a peroxide treatment and protodesilylation generated diol **355** with excellent diastereoselectivity and in 80% yield. Subsequent selenocarbonate formation and radical 5-*exo*-*trig* cyclisation [TTMSS = tris(trimethylsilyl)silane] produced dilactone **356** and set the stage for the remaining oxetane generation via site-specific photochemical desaturation, which is the main highlight of this synthesis. Irradiation of a hexafluoroisopropanol solution of **356** in presence of NBS and catalytic benzophenone promoted the desired bromination/elimination sequence, producing alkene **357** on a gram-scale in a remarkable 82% yield. Finally, stereoselective epoxidation from the sterically more accessible face of the all *cis*-fused tricyclic part of the polycycle, followed by an acid-catalysed epoxide opening enabled the remaining oxetane formation. Thus, (−)-merrilactone A was obtained in a gram quantity and an excellent 23% yield over 13 steps.

#### 4.4 Dictyoxetane

**Scheme 76:**
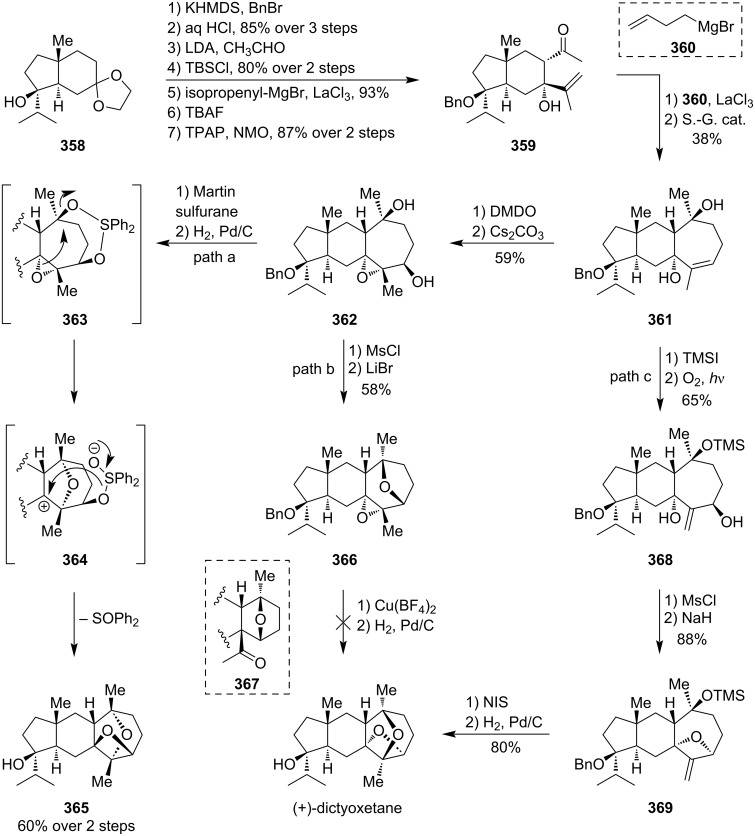
Total synthesis of (+)-dictyoxetane.

This diterpene possessing an unprecedented 2,7-dioxatricyclo[4.2.1.0^3,8^]nonane ring system (Scheme 76) was isolated in 1985 from the brown alga *Dictyota dichotoma* found in the Indian Ocean [25]. Although its biological activity was not reported, the challenging pentacyclic structure made it a formidable target. The first total synthesis was completed by Hugelshofer and Magauer in 2016 (Scheme 76) [138]. It commenced with acetal-protected bicyclic hydroxyketone **358** accessible from commercially available 2-methylcyclopentanone by a known 5-step enantioselective procedure [139]. After initial protection of the alcohol as a benzyl ether, the acetal was hydrolysed and the unmasked ketone was regioselectively deprotonated and alkylated with acetaldehyde, which proceeded exclusively from the bottom face to avoid clashes with the relatively bulky methyl at the ring junction. Subsequent alcohol silylation and La-mediated Grignard addition of an isopropenyl group followed by desilylation and oxidation delivered hydroxyketone **359** in a high yield. Another diastereoselective La-mediated Grignard addition followed by a ring-closing metathesis enabled by the Stewart–Grubbs catalyst afforded the key tricyclic intermediate **361**. The remaining task to construct the tetrahydrofuran and oxetane rings was found to be exceptionally challenging. Although the initial investigation via epoxidation/Payne rearrangement was met with failure, it allowed the authors to discover a remarkable transformation: treatment of epoxydiol **362** with Martin sulphurane (path a) led to tetra-*epi*-dictyoxetane **365**, presumably via cyclic sulphurane **363** formation, epoxide opening, sulphoxide elimination and intramolecular carbocation trapping. Next, the authors attempted a copper-mediated dyotropic rearrangement from polycycle **366** produced by an intramolecular Williamson etherification from diol **362** (path b), but they only observed a ring contraction providing oxanorbornane **367**. Finally, silylation and photooxidation of hydroxyalkene **361** (path c) followed by an S_N_2 substitution generated methylidene-oxetane **369**, which upon iodoetherification and subsequent hydrogenolysis afforded enantiopure (+)-dictyoxetane.

#### 4.5 Dichrocephone B

**Scheme 77:**
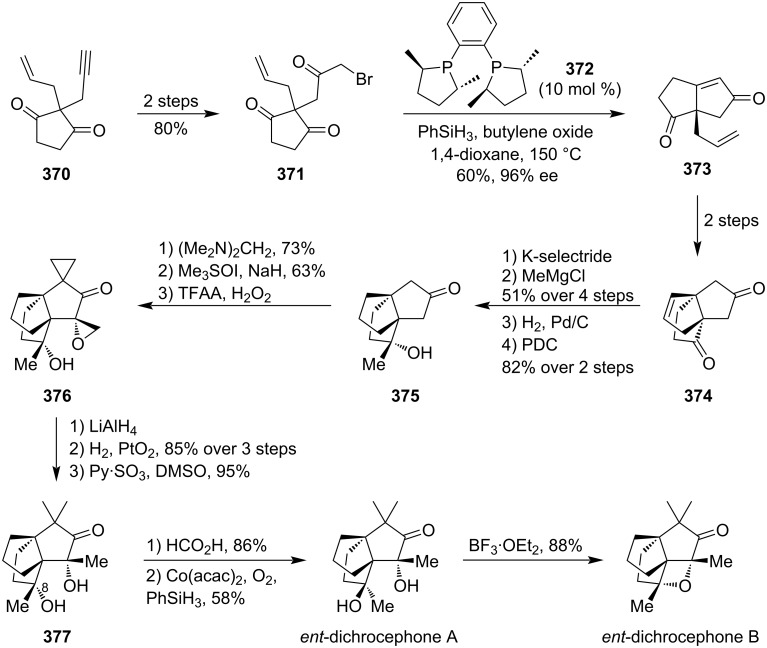
Total synthesis of *ent*-dichrocephone B.

This sesquiterpenoid (Scheme 77) was isolated from the herb *Dichrocephala benthamii* found in China and India and it displays a significant cytotoxicity against HeLa, KB and A549 cell lines [27]. Although the incorrect enantiomeric form was assigned upon discovery in 2013, Tantillo, Christmann and co-workers revised the structure 5 years later based on their enantioselective synthesis (Scheme 77) [140]. Starting from readily accessible 2-allyl-2-propargylcyclopentane-1,3-dione (**370**), they prepared triketone **371** in 80% yield via an alkyne bromination/hydration sequence. Subsequent catalytic asymmetric Wittig reaction provided bicycle **373** in 60% yield and 96% ee, which was then converted to propellane **374** through a conjugate addition of a vinylcuprate and a ring-closing metathesis. A series of functional group manipulations, including a Grignard reaction, yielded hydroxyketone **375**, which upon a double α-methylenation, Corey–Chaykovsky cyclopropanation and OH-directed epoxidation gave spirocycle **376**. Reductive openings of the 3-membered rings and reoxidation of the ketone delivered tricycle **377** which was expected to be dichrocephone A, the biosynthetic precursor to dichrocephone B. However, discrepancies in the NMR spectra strongly suggested that **377** was only a diastereomer of dichrocephone A and a structural revision was necessary: epimerisation of the C8-hydroxy through dehydration with formic acid followed by rehydration under Mukaiyama conditions eventually afforded the correct diastereomer but opposite enantiomer, showing that the originally reported absolute configuration had been determined incorrectly (most likely due to the ECD spectra being calculated for the wrong epimer **377**). Finally, treatment with BF_3_ induced the oxetane formation via dehydrative etherification, producing *ent*-dichrocephone B in 88% yield.

#### 4.6 Mitrephorone A

**Scheme 78:**
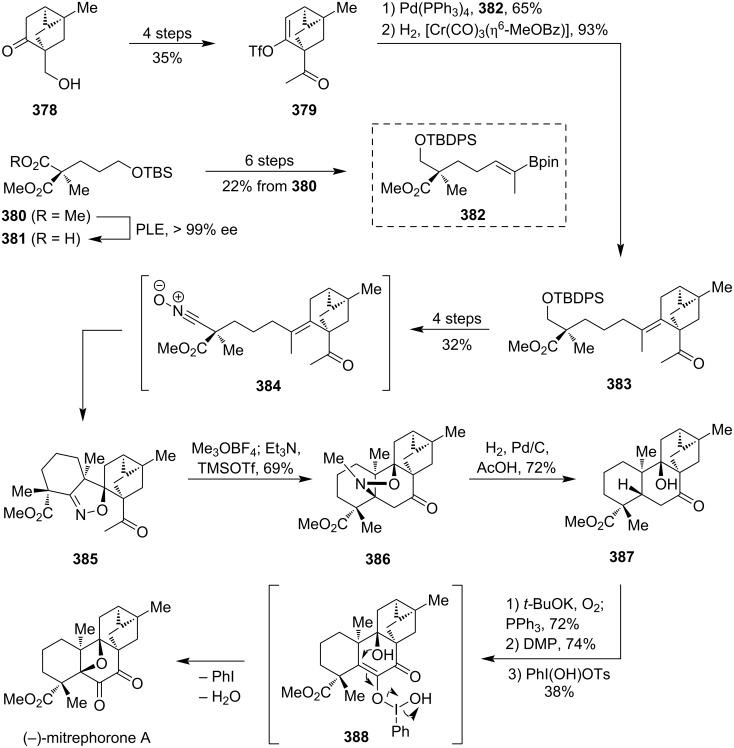
Total synthesis of (−)-mitrephorone A.

Mitrephorone A (Scheme 78) is a trachylobane diterpenoid isolated from the plant *Mitrephora glabra* Scheff. in 2005 and it displays a modest cytotoxicity and antimicrobial activity [26]. The most recent total synthesis of this natural product was reported by Carreira and co-workers in 2020 (Scheme 78) [141]. It commences with a series of simple modifications to the tricyclooctane core **378** whose asymmetric synthesis had previously been reported by the same research group [142]. Then, Suzuki coupling of vinyl triflate **379** with enantiomerically pure vinylboronate **382** [produced via enzymatic desymmetrisation with pig liver esterase (PLE)] followed by a Cr(0)-mediated 1,4-semihydrogenation afforded tetrasubstituted alkene **383** as a single isomer in 60% yield over 2 steps. Subsequent functional group manipulations gave nitrile oxide **384** which upon heating underwent a highly stereoselective dipolar cycloaddition, producing isoxazoline **385** in 32% yield as the only diastereomer. Activation of the cyclic oxime by methylation followed by a Mannich reaction provided isoxazolidine **386**. Then, hydrogenation with Pd/C in acetic acid at an elevated temperature led to β-hydroxyketone **387** via N–O-bond cleavage and reductive elimination of the resulting methylamino group. The remaining task was to install another ketone and to form the oxetane: after α-hydroxylation with dioxygen followed by alcohol oxidation with DMP, the resulting readily enolisable diketone was treated with the Koser’s reagent [143], forging the oxetane ring through a unique oxidative cyclisation (**388**). Thus, enantiomerically pure (−)-mitrephorone A was obtained in 38% yield over 3 steps.

#### 4.7 Taxol

**Scheme 79:**
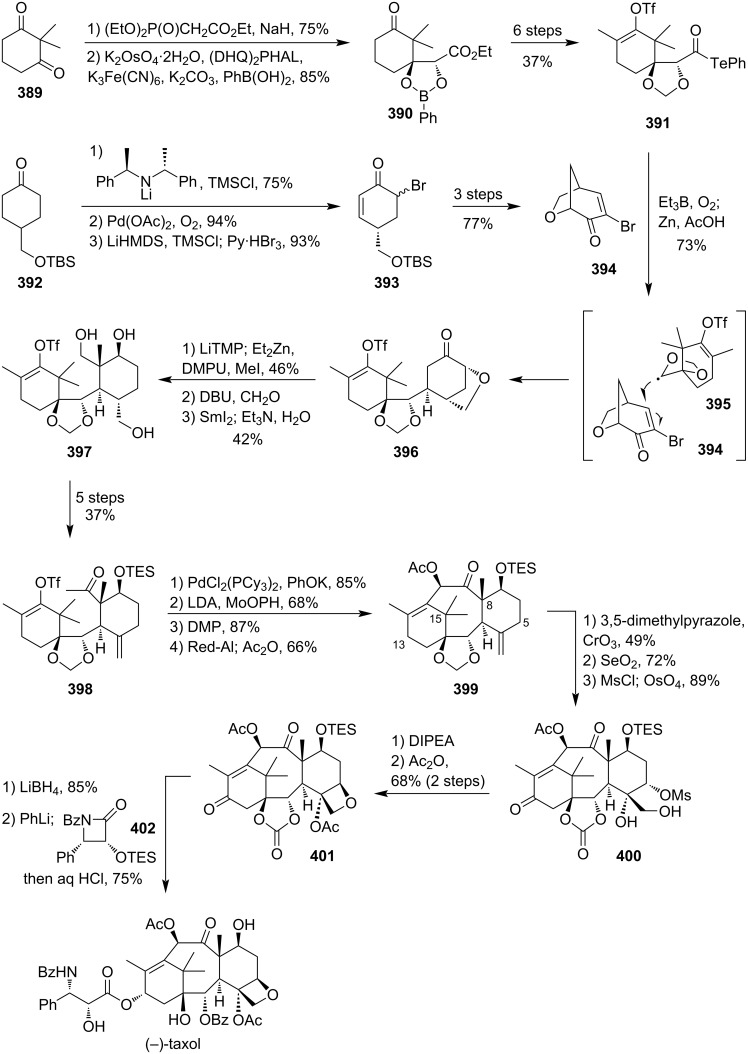
Total synthesis of (−)-taxol.

The structure of this famous taxane diterpenoid (Scheme 79) was reported by Wani et al. in 1971 upon isolation from the stem bark of the western yew tree (*Taxus brevifolia*) [21]. Its highly oxygenated and strained [6-8-6-4] tetracyclic core, containing an anti-Bredt bridgehead alkene and 11 stereogenic centres, quickly drew the attention of many organic chemists, and the desire to develop a total synthesis was further motivated by taxol’s unprecedented ability to promote microtubule assembly resulting in a potent anticancer activity [144]. By the end of 2022, 10 total and 3 formal syntheses have been reported, and these are well summarised in a review written by Li and colleagues [145]. However, in 2023 Inoue et al. disclosed two additional total syntheses [146–147] of which the more recent and more efficient one is briefly described in Scheme 79: commercially available dimethylcyclohexanedione **389** was first subjected to a Horner–Wadsworth–Emmons monoolefination and then to a modified Sharpless asymmetric dihydroxylation in the presence of phenylboronic acid to trap the resulting diol as a boronate ester, thus allowing for an efficient recrystallisation that raised the enantiopurity from 96 to >99% ee. Subsequent standard functional group manipulations delivered telluroester **391** in 37% yield over 6 steps. In a parallel sequence, cyclohexanone **392** was desymmetrised by silylation using a chiral amide base, and the resulting silyl enol ether underwent Saegusa–Ito oxidation and bromination to obtain bromoketone **393**, which was readily turned into bicyclic bromoenone **394** in 77% yield over 3 steps. Following a radical coupling with **391** and reductive elimination of the bromide using Zn/AcOH afforded adduct **396** as a single diastereomer in 73% yield. This coupling process, initiated by ethyl radicals formed by the reaction of Et_3_B with oxygen, was proposed to follow these key steps: homolytic cleavage of the C–Te bond to form an acyl radical, decarbonylation generating electron-rich alkoxy radical **395** and Giese addition to alkene **394** (doubly activated by the carbonyl and bromide) with substrate-controlled stereoselectivity, where the top face of radical **395** is shielded by the dimethyl group and the bottom face of the enone is hindered by the ether bridge. Next, double stereoselective alkylation of the ketone with iodomethane and formaldehyde, proceeding from the α-face to prevent clashes with the bulky cyclohexene ring, followed by Sm-mediated cleavage of the ether bridge and reduction of the ketone delivered triol **397**, which was smoothly converted to methylketone **398** in 5 steps and 37% yield. To forge the strained 8-membered ring, **398** was subjected to a Pd-catalysed intramolecular cross-coupling reaction which, according to the authors, was favoured by the conformational restriction imposed by the spirally fused dioxolane. Subsequent α-hydroxylation of the ketone with MoOPH proceeded well but, due to the bulky β-oriented methyl at C15, delivered the hydroxy from the α-face, so an epimerisation was carried out via oxidation/reduction, and the desired β-alcohol was then acetylated to give tetracycle **399**. Treatment with CrO_3_ in presence of 3,5-dimethylpyrazole regioselectively oxidised the allylic C13 methylene to ketone and the acetalic methylene to carbonate. Another allylic oxidation with SeO_2_ installed an alcohol at C5 opposite the shielding C8-methyl, and, after mesylation, the neighbouring alkene was stereoselectively dihydroxylated with OsO_4_ (again from the face opposite to the C8-methyl) to afford diol **400**. Subsequent intramolecular Williamson etherification promoted by heating in the presence of ethyldiisopropylamine formed the oxetane ring and the remaining alcohol was acetylated, affording pentacycle **401** in a high yield. Next, the sterically more accessible ketone at C13 was stereoselectively reduced from the convex face and then, sequential addition of phenyllithium, β-lactam **402** and dilute HCl transformed the carbonate into benzoate ester, incorporated the β-amino acid side chain and hydrolysed the silyl groups, respectively. Thus, enantiomerically pure (−)-taxol was obtained in 0.055% overall yield over a total of 28 steps.

## Conclusion

Oxetanes are cyclic, highly strained 4-membered monoethers that rarely appear in natural products but quite frequently in the laboratories of medicinal chemists. They are excellent hydrogen-bond acceptors, surpassing other cyclic monoethers and even most carbonyl functional groups. In combination with their relatively high polarity and metabolic stability, they make perfect isosteric replacements for carbonyl and *gem*-dimethyl groups which is why they have become so popular in medicinal chemistry over the last decades.

There are two possible approaches to the synthesis of oxetanes: the first one is a de novo formation of the 4-membered ring, most commonly via Williamson etherification or [2 + 2] cycloaddition. Significant advancements have been achieved in both reactions including the novel photoredox annulation of alcohols with vinylsulphonium triflates or the first highly enantioselective Paternò–Büchi reaction. New methodologies have been developed in the less common strategies as well, for example visible-light-induced 1,5-HAT leading to an unprecedented C–C-bond-forming cyclisation, catalyst-free ring contraction of 2,5-dihydrofurans or generation of phosphonate oxetanones via gold-mediated O–H insertion. Truly inspiring contributions have also been made in the second approach which is based on derivatisation of 3-oxetanone as the principle oxetane building block: alkylation of thiols by 3-aryl-3-oxetanols to provide novel bioisosteres of thioesters, or highly practical and modular syntheses of oxetane-based benzamide isosteres via defluorosulphonylative coupling or Katritzky’s benzotriazole chemistry are among the most remarkable reports.

Besides the applications in drug design, oxetanes are also versatile synthetic precursors, mainly due to their inherent ring strain which allows for ring-opening reactions and ring expansions. This reactivity has been exploited for the synthesis of simple heterocyclic molecules such as 5- and 6-membered cyclic ethers, furans, pyrroles or oxazolines, as well as complex polycyclic heterocycles including benzoindolines, benzolactones, benzodioxepines, dihydroquinolines or furoindolines. Novel catalytic desymmetrisation methodologies for ring opening of 3-substituted oxetanes have also emerged, employing chiral phosphoric acids, bisoxazoline–metal complexes and squaramides. The scope of intermolecular ring-opening reactions has been expanded as well, for example by the first reported generation of synthetically useful radicals from oxetanes, or by an exceptionally mild protocol employing silyl ketene acetals as the nucleophiles.

Finally, it appears that oxetane-containing natural products are quite popular synthetic targets, not only because of their challenging structures but also due to the intriguing biological activities they exhibit. In many cases, several total syntheses of the same compound have been reported, still pushing the limits of synthetic efficiency and ideality further away. One of the most remarkable ones is the eleventh reported total synthesis of merrilactone A by Zhang et al. who managed to produce the complex pentacyclic molecule on a gram scale, as a single enantiomer and in only 13 steps with an outstanding average yield of 89% per step.

Overall, we have come a long way in uncovering the reactivity and physicochemical properties of oxetanes, and it is fascinating to see that new findings are still being reported, even after almost 150 years since the discovery of oxetane. These efforts to develop novel reaction pathways and to increase efficiency of the established transformations seem to be unceasing - quite certainly, we can already feel excited about the upcoming discoveries.

## Data Availability

Data sharing is not applicable as no new data was generated or analyzed in this study.
